# Evolution of Pyrrolysyl-tRNA Synthetase: From Methanogenesis to Genetic Code Expansion

**DOI:** 10.1021/acs.chemrev.4c00031

**Published:** 2024-07-02

**Authors:** Nikolaj G. Koch, Nediljko Budisa

**Affiliations:** † Department of Chemistry, Institute of Physical Chemistry, University of Basel, 4058 Basel, Switzerland; ‡ Department of Biosystems Science and Engineering, ETH Zurich, 4058 Basel, Switzerland; § Biocatalysis Group, Institute of Chemistry, Technische Universität Berlin, 10623 Berlin, Germany; ∥ Chemical Synthetic Biology Chair, Department of Chemistry, University of Manitoba, Winnipeg MB R3T 2N2, Canada

## Abstract

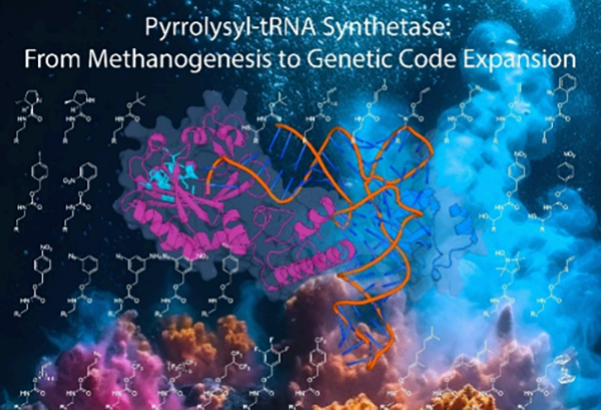

Over 20 years ago, the pyrrolysine encoding translation system was discovered in specific archaea. Our Review provides an overview of how the once obscure pyrrolysyl-tRNA synthetase (PylRS) tRNA pair, originally responsible for accurately translating enzymes crucial in methanogenic metabolic pathways, laid the foundation for the burgeoning field of genetic code expansion. Our primary focus is the discussion of how to successfully engineer the PylRS to recognize new substrates and exhibit higher *in vivo* activity. We have compiled a comprehensive list of ncAAs incorporable with the PylRS system. Additionally, we also summarize recent successful applications of the PylRS system in creating innovative therapeutic solutions, such as new antibody–drug conjugates, advancements in vaccine modalities, and the potential production of new antimicrobials.

## 1 Introduction

Throughout Earth’s history, metabolic innovations, such as the development of methanotrophy, have transformed the carbon cycle from a geological process into a biochemical one.^1^ This transition led to the production of methane as a metabolic byproduct, but it likely resulted in low biomass productivity compared to the modern biosphere. Consequently, the biocatalysts in hydrogen-based chemolithotrophic methane-cycling ecosystems were most probably not very efficient.^2^ The pyrrolysine (Pyl) coding system in protein translation is believed to have originated in such a milieu: in pre-LUCA (last universal common ancestor) progenitor organisms, specifically in a group of hydrogen-dependent methylotrophic methanogens.^3,4^

This coding system is closely tied to methanogenesis, where pyrrolysyl side chains play a catalytic role in a few enzymes. The strong link between methanogenesis and the catalytic function of pyrrolysyl side chains in these enzymes influenced the genetic code in these organisms. As a result, rarely used amber termination codons were reassigned to the specific canonical amino acid Pyl (also known in the literature as 22nd amino acid), resulting in a genetic code found in only a handful of species and proteins. In particular, pyrrolysyl-groups are exclusively incorporated into a few methyltransferases involved in methanogenesis without the need for a high substrate specificity and catalytic turnover.^3,5^

**Figure 1:**
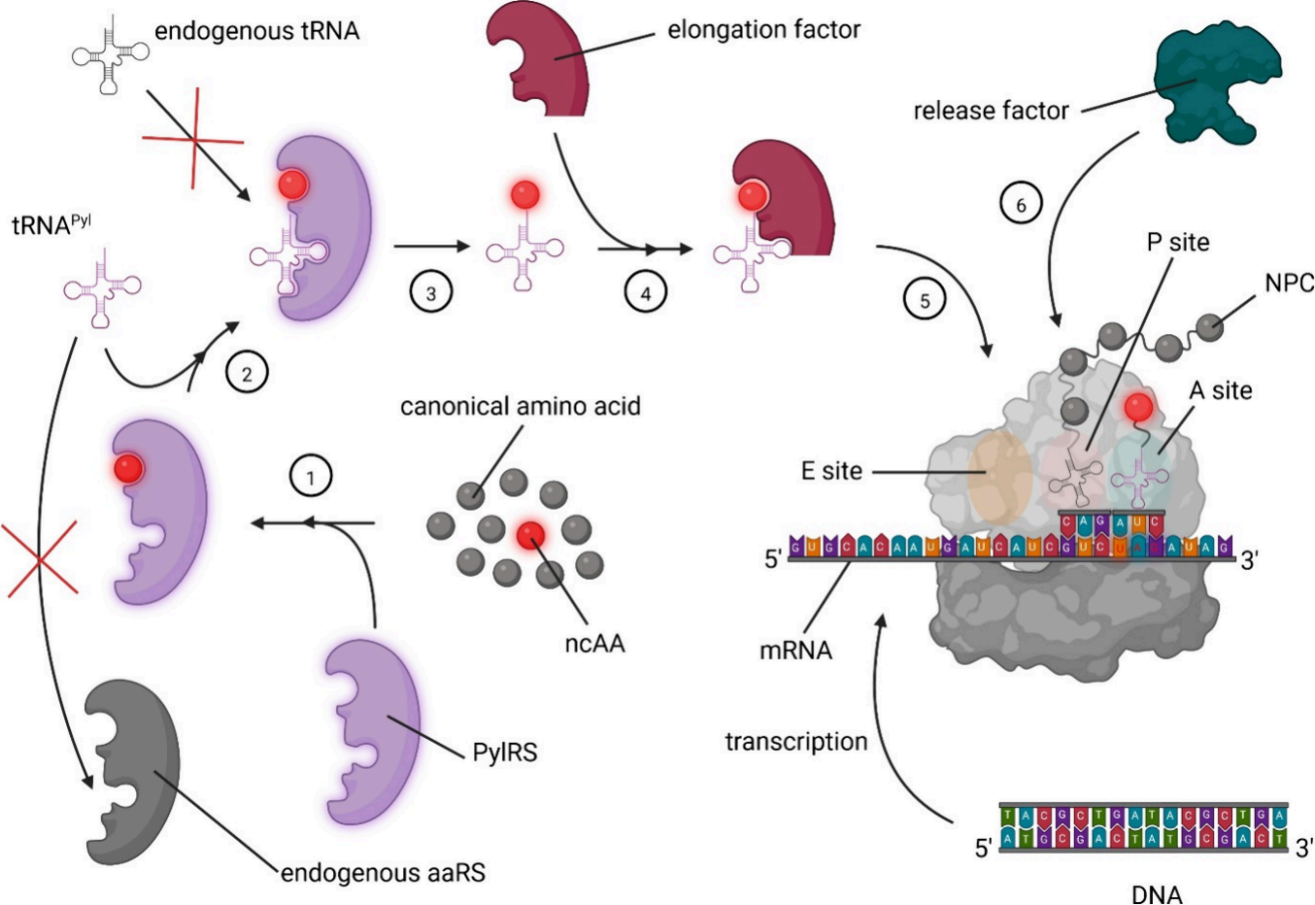
Mechanism of in-frame amber stop-codon readthrough. 1) Activation of a ncAA by PylRS. 2) Binding and aminoacylation (“loading” or “charging”) of tRNA^Pyl^ by PylRS. 3) Aminoacylated tRNA^Pyl^ dissociates from PylRS. 4) Binding of tRNA^Pyl^ to elongation factor. 5) Binding to the aminoacyl site (A site) of the ribosome followed by movement to the peptidyl site (P site) for elongating the nascent polypeptide chain (NPC). Subsequently, tRNA^Pyl^ moves to the exit site (E site) and leaves the ribosome. 6) Translation terminates upon the arrival of a nonamber stop-codon at the A site. At that point, the release factor (RF) binds to the ribosome and initiates release of the polypeptide chain by dissolution of the translation complex.

The Pyl-insertion system with its dedicated pyrrolysyl-tRNA synthetase (PylRS), which plays a rather marginal role in nature, became one of the most important tools for genetic code expansion (GCE). Indeed, the introduction of GCE technologies has revolutionized the life sciences and all related fields. The ability to make proteins containing new-to-nature noncanonical amino acids (ncAAs) has vastly expanded the chemical and functional space of proteins. This expansion has led to a huge increase in applications in basic and applied sciences, including medical therapies.^6−21^ The specific incorporation of ncAAs relies on orthogonal translation systems (OTSs) consisting of engineered orthogonal aminoacyl-tRNA synthetase/tRNA pairs. The most common site-specific incorporation method is amber stop codon suppression (SCS). In SCS, the ncAA is incorporated in response to an in-frame stop codon placed at a predefined position in the coding sequence of the target protein that is expressed ribosomally either *in vivo* or *in vitro* (Figure 1). While there are a few different OTSs,^22,23^ in terms of substrate diversity and functionality, the most popular systems are PylRSs-based OTSs which will be the focus of this Review. There is a relatively new class of chimeric PylRS enzymes that looks very promising, but since addressing the PylRS system is already a major undertaking, we will also exclude these systems.^24^ At least 342 substrates have been incorporated into proteins with the PylRS OTS, including some α-hydroxy acids and nonalpha amino acids.

One reason for the popularity of the PylRS certainly is the natural orthogonality of this system in all three domains of life and, compared to other OTSs, the ease with which it can be constructed for different substrate recognitions while retaining its original orthogonality.^25−27^ The ability to tolerate anticodon mutations to recognize codons other than the amber stop codon undoubtedly also contributes to the success of this system.^28−32^ This Review provides an update and useful source of all the substrates that can be incorporated into proteins with the PylRS system and the applications for which these ncAAs can be used. Since the number of substrates that can be incorporated with the PylRS system is over 340, we cannot go into detail on each individual ncAA. In addition, we summarize the experience from all enzyme engineering campaigns and add considerations that are important for the design of new substrate recognitions and improvements of the *in vivo* efficiency of PylRS. Finally, we will present some highlights of recent GCE applications and future directions.

The Review starts with an introductory overview encompassing the history, function, and different classes of PylRS OTS (section 2). This is followed by an overview and discussion strategies on how to best engineer the PylRS substrate specificity (section 3) and enhance ncAA incorporation efficiency (section 4). Subsequently, potential avenues for expanding the accessibility of PylRS OTS to a wider scientific community are explored, particularly by coupling the current systems to endogenously produced ncAAs (section 5). Section 6 provides a comprehensive survey of existing literature on the biological applications of PylRS OTS with an emphasis on therapeutic applications. Following this, limitations associated with encoding multiple ncAAs are addressed, along with strategies to overcome them and ideas for further improvement (section 7). Finally, the Review concludes with a reflective overview of the field, offering insights and suggestions crucial for advancing not only PylRS OTS but also all GCE systems in the future (section 8).

## 2 The PylRS System

In 1998, Krzycki and co-workers serendipitously discovered that certain methyltransferases important for methane metabolism in *Methanosarcina* contain an in-frame stop codon.^33,34^ In 2002 Krzycki, Chan and colleagues elucidated that this stop codon encoded the 22nd amino acid Pyl.^35,36^ In 2004, a landmark year for the field, the pioneering efforts of several research groups, including those of Söll, Wood, Crain and Chan, and Krzycki, brought our understanding of the intricate mechanisms underlying the Pyl coding pathway a significant step forward. Independently, these teams demonstrated the existence of a specific aaRS (PylRS) that is responsible for the exclusive loading of the tRNA (tRNA^Pyl^) with the specific noncanonical amino acid pyrrolysine.^37,38^

Methane-producing microorganisms are thought to be among the earliest cellular life forms that colonized various anaerobic habitats on our planet.^39^ The discovery of a PylRS system distinct to the ones found in bacteria suggests that the origin is most likely in a pre-LUCA progenitor, or close LUCA descendant, and later spread through horizontal gene transfer (HGT).^5^ Most likely, the metabolic needs of methanogenic archaea living in extreme habitats were the driving force for the natural expansion of the genetic code (i.e., reassignment of the Amber codon to Pyl).^3^ The addition of Pyl could be a recent evolutionary event or a “fossil” derived from a pre-LUCA lineage representing a hypothetical extinct fourth domain of life, as speculated by Fournier and associates.^3,40^ In 1976, Jensen proposed that ancient enzymes were less efficient but had very broad specificity as a kind of trade-off.^41^ The remarkable substrate tolerance (promiscuity) of the wild-type enzyme, in activating substrates with different amino acid side chains and several carboxylic acids not containing any amino group,^42^ supports the view that the PylRS is an evolutionary very old enzyme. Given that this aminoacyl tRNA synthetase (aaRS) is exclusively employed for incorporating Pyl into three genes (*mttB*, *mtbB*, and *mtmB*) at a single site,^43^ it appears that there has been no significant evolutionary pressure to optimize this enzyme for efficiency. Moreover, because the considerable size of Pyl compared to canonical amino acids (cAAs) and its distinct nature from most metabolites, the evolution of highly specific chemical interactions for exclusive recognition of Pyl has been unnecessary.^44^ A more in-depth discussion on this topic will follow in section 3.

**Figure 2:**
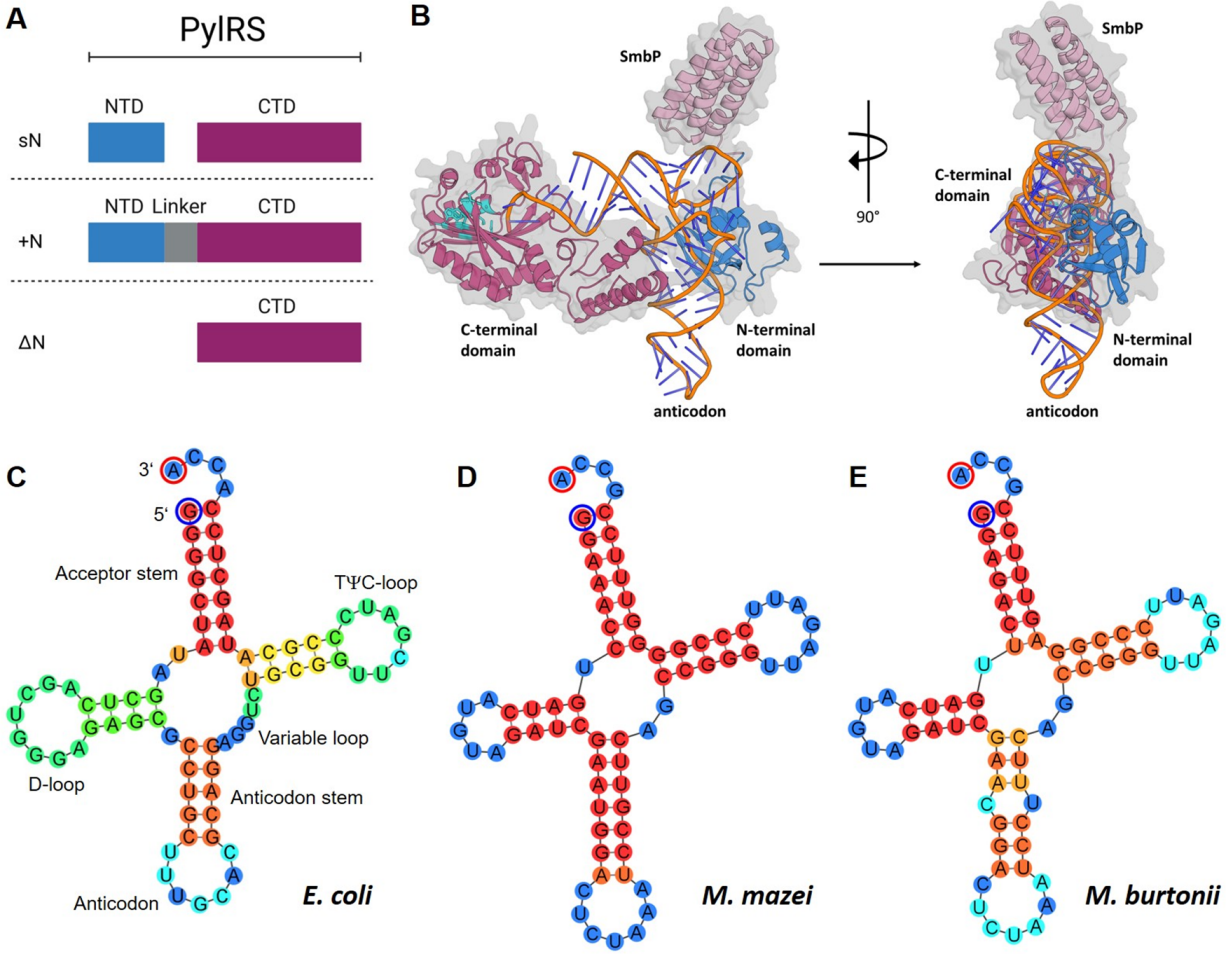
A) Schematic representation of the three PylRS classes. NTD = N-terminal tRNA-binding domain; CTD = catalytic domain. B) Cartoons illustrating the tRNA^Pyl^ recognition mechanism of *M. barkeri* PylRS with the N-terminal fused small metal binding protein (SmbP = light pink) domain to enhance in-cell solubility.^64^ The 3D model structure (cartoon representation) was calculated using ColabFold (C-terminal domain = purple, N-terminal domain = light blue).^65^ The model alignment involved the N- and C-terminal domains, aligning them with the corresponding domains of *Methanosarcina mazei* (*M. mazei*) (PDB ID 5UD5) and *D. hafniense* (PDB ID 2ZNI) bound to tRNA^Pyl^.^45,55^ The conserved active site residues are shown as sticks (cyan). For clarity and because it is unstructured, the linker region has been omitted. The N- and C-terminal domains of PylRS recognize the tRNA^Pyl^ in the anticodon stem region. Unlike canonical tRNAs, tRNA^Pyl^ has only a small variable arm, explaining its orthogonality to all other canonical tRNA/aaRS pairs. Additionally, the anticodon is not involved in the recognition mechanism. C, D, and E) Comparative analysis of *E. coli* tRNA^Ala^ and two tRNA^Pyl^ variants.^27,66^ For the illustration, *E. coli* tRNA^Ala^ was selected as a representative example of a canonical tRNA. The folding predictions were conducted using *Geneious* (version 7.1.9) employing the *ViennaRNA* Package.^67^ The color code indicates the predicted binding strength.

While the PylRS system was originally found in only a few organisms, recent studies show that it is widespread across all domains of life.^4,5,45,46^ All known PylRS OTSs can be divided into three classes: PylSn+PylSc (sN), PylSn–PylSc fusion (+N), and ΔPylSn (ΔN) (Figure 2A) with a total of number of 79 + N, 66 ΔN, and 204 sN sequences,^46^ with the +N class the only one where the N- and C-terminal domain are connected. Investigations conducted with the +N PylRS system revealed that there are only two essential components required to encode Pyl in response to the amber codon: The PylRS and the tRNA^Pyl^. These works also showed that this system can be easily transferred to other organisms, e.g., *E. coli*.^37,38,47^ The same is also true for the ΔN class as the wealth of newer publications using this class shows.^46,48,49^ The sN class gives a mixed picture in terms of transferability to *E. coli*. In the best-case scenario, the systems that are transferable to *E. coli* resulting in reduced growth indicating some toxicity. Deleting the gene for the separately encoded N-terminal domain restored normal growth.^46^ It is not clear what role the separately encoded N-terminal domain (NTD) plays since these system are functional without it.^50,51^ They are even sometimes more functional *in vivo* without this domain.^50^

Even though the two non-+N classes are mutual orthogonal, they recognize their cognate tRNA^Pyl^ with similar mechanisms. They recognize the tRNA^Pyl^ at the acceptor stem but the tRNAs possess different identity elements leading to mutual orthogonality. Interestingly, these identity elements correlate with different tRNA^Pyl^ rigidities for each PylRS class.^52^ For the +N PylRS class, the NTD is crucial for tRNA^Pyl^ recognition and binding *in vivo*, and the C-terminal domain (CTD) is essential for catalytic aminoacylation. These domains are connected by a linker. It is noteworthy that the linker can vary drastically in size within certain genera, specifically *Methanosarcina*, and the amino acid composition may differ substantially among different genera.^27^ The CTD contains a conserved catalytic core with a Rossmann fold characteristic of class II aaRS.^53^ Structural phylogeny analyses also support this assignment, although tRNA^Pyl^ recognition is unique.^54^ It is this unique binding mode that makes the PylRS OTS orthogonal in all domains of life. Most aaRSs recognize their cognate tRNAs at the anticodon or acceptor stem.^53^ The tRNA^Pyl^ is different: because it has almost no variable loop and an elongated anticodon stem, the anticodon arm and T-loop have close interactions with the PylRS and function as recognition elements (see Figure 2B).^55^ This also explains the “blindness” of the anticodon of tRNA^Pyl^,^45^ which has been exploited for the reassignment of proteome-wide sense codons and assignment of newly created codons in a semisynthetic organism.^28,30,56,57^ Together, the structural and evolutionary evidence supports the notion that the C-terminal PylRS domain originated from an ancient PheRS after the occurrence of LUCA.^5,54^ In contrast, the NTD lacks any identified structural or sequence homology with known RNA-binding proteins.^4,58^ Although the precise physiological function of the NTD remains unclear, its affinity for tRNA^Pyl^ implies a capability for recruiting tRNA^Pyl^.^55,58^ Considering the variations in linker sizes and compositions, it is reasonable to suggest that the NTD can tune the tRNA^Pyl^ affinity in conjunction with the linker, as supported by available data.^59^ The hypothesis that the +N class exhibits a higher tRNA^Pyl^ affinity than the ΔN class is supported by kinetic data.^4^ Newer results also suggest that the NTD stabilizes the correct tRNA^Pyl^ geometry necessary for aminoacylation.^52^

In general, the +N class is the most prevalent, primarily due to historical reasons as it was the first discovered PylRS class. Additionally, this class has often higher *in vivo* activity compared to ΔN variants especially in mammalian cells, resulting in either higher yields of target proteins or the ability to work with lower working concentrations of ncAAs.^4,60−62^ These factors have contributed to the widespread use of the PylRS +N class. To avoid overgeneralization, we want to emphasize that also the plasmid setup (promotors, origin of replication, antibiotic resistance, copies of PylRS genes) is very important for the *in vivo* performance. Recently, there have been a few reports showing very efficient ΔN variant systems in bacteria.^49,63^ The inherent diversity in PylRS sequences led to the identification of mutual orthogonal PylRS OTS pairs,^61,62^ and further engineering enhanced this orthogonality, resulting in five PylRS OTS which are mutual orthogonal.^46^

## 3 Engineering New ncAA Recognition for the PylRS

Before diving into the details of how to encode new ncAA recognitions, it is essential to establish the theoretical constraints of encoding certain ncAAs. In theory, any ncAA capable of entering the host cell and being efficiently discriminated against by the endogenous aaRSs of the target host is considered possible to encode. However, if discrimination is ineffective or inefficient, the consequence could be severe toxicity for the host cell, potentially leading to the host’s demise in extreme cases.^68^

In the realm of enzyme engineering for new substrates, two pivotal attributes play a crucial role. First, in the best case, the target enzyme ideally possesses low levels of the desired activity or is closely aligned with the new intended activity.^69,70^ This sets an optimal foundation for successful modifications. Second, there is a requisite for sufficient stability, acting as a buffer against destabilizing mutations necessary for remodeling the active site.^69,71−73^ Obviously, the extent of this prerequisite is contingent on the number of mutations required to encode the new function. The combination of structural, thermodynamic, and kinetic properties is a distinctive feature of enzyme structures, which represent a typical trade-off between structure and function. This trade-off is often about gaining a new function at the expense of protein stability.^74^

Since the survival of every organism depends on very high fidelity of its translational machinery,^53,75,76^ the first part of aaRS engineering poses a notably greater challenge than that of other enzymes. In general, aaRSs exhibit a selectivity that is 2–3 orders of magnitude higher than most other amino acid-utilizing enzymes.^77^ That gives an idea on how many mutations are required, in comparison to other enzymes, to redirect the substrate recognition of an aaRS toward a radically different substrate.

The complexity of this challenge varies depending on the specific aaRS under consideration. Some aaRSs have multiple layers of functional elements to ensure the necessary fidelity, such as editing domains in IleRS.^75^ This implies that the number of mutations needed to alter the substrate specificity is aaRS specific. More precisely, it correlates with the number of elements required for an aaRS to discriminate a cAA among the other 19 cAAs and all other small molecules within an organism.^75^

Moreover, the chemical similarity between the desired ncAA and the original substrates also influences the number of mutations needed. The more distinct they are chemically, the greater the number of mutations required for successful alteration. Considering all these constraints, it is not coincidental that the first efficient *in vivo* GCE system was derived from the *Methanocaldococcus jannaschii* tyrosyl-tRNA synthetase (*Mj*TyrRS). Note that the first, but inefficient, OTS was created by Furter derived from a yeast TyrRS.^78^ This choice was deliberate due to specific features of *Mj*TyrRS that made it amenable to engineering. Notably, the two specific Tyr OH interactions of the *Mj*TyrRS (Y32 and N158) could be abolished, and crucially, this aaRS lacks an editing domain.^77,79,80^ Furthermore, the hyperthermophilic origin of *Mj*TyrRS endowed it with the necessary stability to tolerate all the mutations needed to change the substrate and anticodon recognition. In contrast, the attempts to use an *E. coli* tyrosyl-tRNA synthetase (*Ec*TyrRS) were considerably less successful, particularly in terms of the number of ncAAs incorporable with this system.^73^

With the PylRS OTS, no mutations for orthogonalization were needed. The wild type PylRS could be readily used to incorporate 36 ncAAs, α-hydroxy acids,^42,81^ and even an oxazole containing amino acid.^82^ This promiscuity makes it a superior candidate for OTS engineering in comparison to the *Mj*TyrRS or most other developed OTSs.^23^ Empirically, this is very well supported. For example, the *Mj*TyrRS,^83^ the *E. coli* leucyl-tRNA synthetase (*Ec*LeuRS),^84^ or methionyl-tRNA synthetase *Ec*MetRS^85^ all need far more mutations to recognize even slightly altered substrates while maintaining orthogonality compared to PylRS.^86^ Even with these mutations, the substrates often retain a significant resemblance to the native ones. To create an efficient *Mj*TyrRS, for example, computational models are often required to identify up to 10 mutations needed. This complexity makes the engineering of these enzymes far more complicated, reducing the likelihood of discovering the desired enzyme.^87,88^ In contrast, the PylRS OTS typically requires only two to four mutations to drastically alter the substrate recognition, shifting from Lys to Phe, Trp, His, and even small aliphatic analogs. We estimate that the number of substrates encodable with this system is now three-times larger than with all other systems combined (∼100 vs ∼340).

We attribute this remarkably unique behavior to the fact that the PylRS never underwent evolutionary optimization to become a specialist enzyme; instead, it has retained a more generalist nature as discussed earlier.^69^ The PylRS appears to be an ancient enzyme, still exhibiting properties associated with its ancestral state, and these traits have undoubtedly contributed to the wide adoption of this system. Interestingly, some of these ancient traits are generally desirable, as evidenced by the field of enzyme engineering known as ancestral sequence reconstruction (ASR) which is trying to exactly reconstruct these ancient enzymes to harness their properties.^89,90^ In the case of PylRS, the GCE community was fortunate to have such an enzyme without the need for extensive ASR efforts.

Lower aaRS stability can lead to a reduced in-cell abundance of correctly folded and active enzyme, consequently resulting in decreased OTS performance.^73,91^ Unfortunately, it appears that PylRS is marginally stable under standard cultivation conditions in *E. coli*,^92^ a feature that may also be partly reflected in its low *in vitro* solubility.^54,93^ This instability is even more pronounced in the *M. barkeri* PylRS (*Mb*PylRS) variant than in the *M. mazei* PylRS (*Mm*PylRS).^44,55^ As mentioned earlier, having a more thermostable PylRS could be advantageous for engineering PylRS OTS to encode even more diverse set of ncAAs. Although there has been an attempt to elucidate the performance of thermophilic PylRS,^92^ it is asserted that the PylRS they determined as thermophilic (*Mt*PylRS) is, in fact, not thermophilic. This is apparent in the phylogenetic tree they provided in Figure 1 of their work, a point discussed in a previous publication.^27^ As of now, the only known thermophilic PylRS is from *Methermicoccus shengliensis*, belonging to the ΔN class, with an optimal growth temperature (OGT) of 65 °C.^94^

In general, the interplay between enzyme fidelity (or its inverse property, promiscuity) and the concentration of ncAA is frequently overlooked or, at the very least, not actively addressed in the field of OTS engineering. The fidelity of an aaRS signifies the enzyme’s ability to discriminate for one cAA within a given set of molecules. On the other hand, the error rate of an aaRS indicates how frequently a misactivation of a noncognate cAA or ncAA will occur within a given time frame. The time dependency is crucial; an increase in the concentration of noncognate cAAs or ncAAs enhances the likelihood of these molecules visiting the enzyme’s active site, leading to an elevated error rate. This property can be strategically exploited to identify variants which display at least some activity for the desired ncAA. Subsequently, these variants can be further optimized via directed evolution. In a specific case, we employed 10 mM ncAA concentration as default, which is significantly higher in comparison to in-cell cAA concentrations, to find PylRS variants that can activate small aliphatic ncAAs.^25,26^ Notably, this exploration was conducted without the need to construct a PylRS active-site library, employing a purely rational approach. Ultimately, by combining the rational approach with site-saturation mutagenesis (SSM), we found six variants capable of encoding over 20 new ncAAs (**284**–**306**, see Figure 8).^26^ It is noteworthy that in many PylRS engineering campaigns, the ncAA concentration is neither varied nor are attempts made to systematically rationalize the chosen concentrations. Although some studies default to high ncAA concentrations, the reasons behind this choice are rarely discussed or clarified.^46,62,95^

We were the first to address the question of which starting point (in terms of organismal origin) might be the most advantageous when beginning to engineer PylRS for new substrates.^27^ From a protein engineering perspective, it is plausible to start with the most promiscuous variant.^69^ Since we could not screen all known PylRS sequences (over 300),^46^ we made rationalizations and capitalized on a particular class of enzymes: the psychrophilic (cold loving) ones. These enzymes are recognized for their increased flexibility, essential for maintaining activity at low temperatures. This heightened flexibility indicates a greater likelihood for these enzymes to adopt various conformations within a given time frame compared to their nonpsychrophilic homologues, ultimately resulting in higher promiscuity. However, a drawback of psychrophilic enzymes is their tendency to be unstable at normal laboratory cultivation conditions.^96−98^ Nevertheless, in our screening, we identified a highly active psychrophilic PylRS from *Methanococcoides burtonii* (*M. burtonii*). Additionally, our improved *M. barkeri* variant exhibited notable promiscuity.^64^ In summary, there is good reason to believe that these two PylRS will deliver a desired target PylRS substrate specificity with a higher probability than using the other PylRS homologues as starting points.^27^

In this Review, we have summarized that the PylRS system has been engineered to incorporate over 340 substrates. It is noteworthy that the majority of ncAAs incorporated using the PylRS system are relatively long-chained and/or bulky Pyl or Phe analogs. Smaller ncAAs constitute a minority, with a more recent class of small aliphatic ones now also accessible (**284**–**306**).^26^ Additionally, we emphasize the remarkable capability to engineer the PylRS OTS to discriminate between α-hydroxy acids and ncAAs, leading to valuable applications (see section 6).^99^

## 4 Engineering the PylRS for Increased *In Vivo* Efficiency

The ideal OTS would possess both high catalytic efficiency and sufficient versatility or promiscuity, making it amenable to easy engineering with only a few mutations. While the known PylRS variants fulfill the latter criterion, they often lack the former. Enhancing efficiency is crucial to further increase the utility of the PylRS OTS, establishing it as a robust research tool or a routine method for achieving high protein yields to facilitate applications.^100^

**Figure 3:**
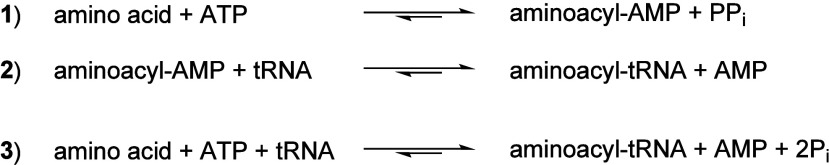
Aminoacylation reaction of tRNA. This reaction cascade is catalyzed by an aaRS. Driving force of the reaction is the pyrophosphate (PPi) hydrolysis. Hydrolysis of PPi is not depicted. 1) Activation of the amino acid. 2) Transfer of the amino acid to the tRNA. 3) Overall chemical reaction. Nucleophilic attack at the α position of ATP displaces PPi and transfers adenylate (5′-AMP) to AA forming aminoacyl-AMP (the reaction is called adenylation in nonribosomal peptide synthesis). The PPi is further hydrolyzed by inorganic pyrophosphatase to two Pi (−20 kJ/mol). This energy release constitutes the driving force of the reaction.

The activation and transfer of an amino acid to the cognate tRNA is a two-step process (Figure 3).^77^ There are generally two classes of aaRS and each has its own rate limiting step.^101^ To ensure meaningful comparisons, it is advisable to focus on overall kinetic values within each aaRS class. In class II aaRS, the rate limiting step is normally the amino acid activation step. In this context, the PylRS generally performs between 1 and 3 orders of magnitude worse than canonical aaRS.^44,102−104^ For example, the *k*_cat_ of His activation by the *E. coli* histidyl-tRNA synthetase (*Ec*HisRS) is 130 s^–1^, while the *Mm*/*Mb*PylRS is 0.1–0.3 s^–1^.^44,105^

In assessing *in vivo* efficiency, the affinity of the cognate tRNA is also crucial. The PylRS/tRNA^Pyl^ exhibits similar *K*_m_ values to the *Mj*TyrRS/tRNA^amber^. Unfortunately, these values are 2 orders of magnitude higher than those observed for canonical aaRS/tRNA pairs.^80,106,107^ Limited data are available for comparing different PylRS/tRNA^Pyl^ pairs across different PylRS classes. The scarce available data suggest that, for the ΔN PylRS class, all kinetic parameters are inferior to those of the +N class. Interestingly, the ΔN PylRS class partly compensates for this by achieving a higher in-cell abundance due to better solubility.^4^

Many efforts have been dedicated to progressively enhance the efficiency of both wild-type and engineered +N PylRS systems. These endeavors can be broadly categorized into three classes. First are improvements of regulatory elements of transcription and translation factors and cellular aspects not directly associated with either the aaRS or tRNA of the OTS. These include optimizing OTS plasmid copy number and promotor strength of aaRSs and/or tRNA genes,^108,109^ optimizing sequence context around the target codon^110^ and elongation factor TU engineering.^111^ Second, strategies aim to liberate codons to avoid competition with release factors by creating quadruplet codons,^112^ liberating existing ones by strain engineering,^113−115^ or creating new ones with synthetic nucleotides.^56,116^ While each of these approaches circumvents competition with the termination machinery, they create new drawbacks. Thus, it has to be carefully scrutinized to assess the potential benefits of these approaches in specific cases. Third was the active engineering of the OTS components, such as tRNA^Pyl^^117−119^ and the PylRS. As discussed above, PylRS is an ancient enzyme with corresponding low *k*_cat_ values,^4,44^ compared to most modern enzymes.^44,120^ Therefore, one might have expected it to be the number one target for improving *in vivo* incorporation efficiency.^100^ While there have been some attempts in this direction, much of the focus was diverted toward expanding the range of substrates that can be incorporated. For instance, we tried to increase the *in vivo* efficiency of *Mb*PylRS by (partly) by addressing the solubility issues by adding an N-terminal solubility tag to *Mb*PylRS. The best variant, containing the small metal-binding protein tag (SmbP) from *Nitrosomonas europaea*, increased the efficiency roughly 5-fold for a mutant PylRS.^64^ This solubility tag also improved the performance of wild type *Mb*PylRS. In another rational approach, Cho and co-workers identified mutations for *Mm*PylRS that reduce in-cell cleavage of the NTD. Interestingly, their data suggest that the N-terminal cleavage is triggered by the tRNA^Pyl^ from *M. mazei* itself.^121^

As for a semirational approach, a classic directed evolution method with error-prone PCR to diversify the entire *Mb*PylRS gene was used, followed by consecutive selection.^122^ Primarily NTD mutations were discovered that improved the *in vivo* incorporation efficiency of **109** by 2.5-fold.^122^ This improvement could imply an enhancement in the *K*_m_ for tRNA^Pyl^, given that the NTD is responsible for tRNA^Pyl^ recognition. However, such mutations should generally enhance the performance for other substrates as well, which is not consistently observed.^123^ On the other hand, that also suggests that the NTD is not purely responsible for tRNA^Pyl^ recognition.

The last two semirational attempts utilized continuous (PACE)^107^ and noncontinuous (PANCE)^63^ phage-assisted directed evolution methods. In the PACE approach, the authors used a chimeric PylRS (chPylRS) consisting of the NTD of *M. barkeri* and the CTD of *M. mazei*. The rationale behind this choice, given the known higher insolubility of the *M. barkeri* NTD compared to *M. mazei*, is unclear and not further elaborated in the publication.^54^ The outcome was an improved variant for BocK (**3**) containing four mutations in the NTD, typically suggesting enhancement in solubility and/or tRNA^Pyl^ affinity. However, they also reported a remarkable 45-fold relative catalytic efficiency increase.^122^ This exceptional increase in catalytic efficiency for four mutations^124^ translated into a moderate 1.5-fold increase of *in vivo* target protein yield when one ncAA was incorporated but was a bit higher for the incorporation of three ncAAs (5-fold).

At first glance, the discrepancy between the *in vitro* and *in vivo* data might appear surprising. However, this can be explained by the use of a standard BL21(DE3) strain, where cells have constant competition between the termination and OTS machinery. Particularly release factor 1 (RF1) is responsible for that. In RF1-containing strains and using one stop codon in a target gene, a substantial increase in catalytic efficiency is required for the mutations to translate into a detectable increase in the target protein readout. Unfortunately, using two stop codons sets too stringent conditions, also impeding detection. This phenomenon was elucidated in detail in our most recent study.^27^ When using the B-95.ΔA strain and reporter proteins with three and five stop codons, the difference of *in vivo* efficiency was far easier to detect because the detectable window was bigger. The fold changes in the B-95.ΔA strain, correlating to the *in vivo* efficiency, were significantly higher. Basically, using RF1-deficient strains increases the dynamic range of the readout signal, which is highly advantageous. Given that typical directed evolution campaigns involve multiple rounds of mutations with rather modest improvements in each round, we strongly recommend using RF1 deficient strains for efficiency improvement campaigns. This leads to a broader window where improved variants can be detected, thereby increasing the likelihood of a successful directed evolution campaign. This broader window of course also exists if a selection system is applied. Unfortunately, a drawback in the PACE approach is that several grams of ncAA are needed, making it economically unfeasible for many substrates. This could be somewhat alleviated with a miniaturized approach and now only a few 100 mg are needed.^125^ In the PANCE approach, a PylRS from *Methanomethylophilus alvus* (*M. alvus*, *Ma*PylRS), a ΔN class PylRS, was used. The outcome was a variant carrying three mutations, exhibiting between 1.2- and 2.5-fold more activity.

It is likely that an increased PylRS efficiency for a given substrate will also result in increased substrate specificity. In our efforts, we aimed to find a variant with increased PylRS efficiency and reduced substrate specificity, or at least no increased substrate specificity. While a PylRS with increased efficiency is generally valuable for higher protein yields more robust handling, we hypothesized that a variant with increased promiscuity would also be very useful when trying to engineer the PylRS for new substrates. In pursuing this goal, we took a novel approach by harnessing the properties of psychrophilic enzymes. We screened several psychrophilic PylRSs and identified an exceptional variant from *M. burtonii* (*Mbur*PylRS).^27^ As mentioned earlier, psychrophilic enzymes are often not only more promiscuous but also catalytically more efficient when stable at the desired cultivation temperatures.^96−98^ We demonstrated that a mutant *Mbur*PylRS can recognize substrate **303** so efficiently that it leads to wild-type reporter protein levels, even for multiple in-frame installations. This marked the first instance of reporting such an efficient mutant PylRS that can recognize an ncAA that is substantially different from Pyl-analogs.

In conclusion, despite directed evolution being a standard method for improving the catalytic efficiency of enzymes,^126,127^ it has not been extensively applied to improve the PylRS OTS. When performed, only a small number of mutations have resulted in substantial *in vivo* improvements. Considering the principle of diminishing returns when evolving an enzyme, and the fact that PylRS is an ancient enzyme, there is a significant potential for efficiency improvements.^124^ For example, previous directed evolution efforts of other enzymes have involved up to 14 rounds of mutations targeting the enzyme, resulting in total of 24 mutations to obtain significantly improved variants, underscoring the substantial room for improvement in PylRS.^128^ Data supporting this hypothesis are from a study where an enhanced chimeric PylRS/PheRS with eight mutations led to a 5-fold increase in *in vivo* efficiency.^129^ Importantly, all directed evolution attempts for PylRS were performed in *E. coli* strains containing the RF1. It can be assumed that the catalytic efficiency of these PylRS was far more improved than observed *in vivo* because of the competition within the translation machinery, as seen in the PACE approach.^107^ This factor should be taken into account when designing future *in vivo* directed evolution campaigns.

The previous discussion primarily focused on kinetic parameters of the PylRS, which are correlated to some extent with the *in vivo* efficiency. While most insights above are from studies performed in *E. coli*, the situation in eukaryotic cells appears to be slightly more complicated. Although enzyme efficiency certainly impacts the performance in these hosts, the presence of nuclear localization signals (NLS)^130^ in the NTD of most +N class PylRS introduces an additional layer of complexity.^131^ The NLS appears to induce transportation to the nucleus, thereby reducing *in vivo* performance. Nikić and co-workers where the first to show that a nuclear export signal (NES) could improve the performance to a certain degree by shuttling a portion of PylRS back to the cytosol.^131^ This improvement was observed in HEK and COS cells. Since their initial study, it has been demonstrated that the NES-tag strategy works in a rodent ND7/23 neuroblastoma cell line,^132^ primary mouse cortical neurons,^132^ and also in *Caenorhabditis elegans* (*C. elegans*).^133^

As mentioned earlier, there was an attempt by Hu and co-workers to use thermophilic PylRS to improve GCE in mammalian cells.^92^ The most likely explanation for the different performance of their constructs is not necessarily due to differences in thermal stabilities but rather because their constructs exhibit different localization distributions within the cells. Enzyme localization is a major factor for the efficient function in mammalian cells, particularly for aaRS.^134^ Reinkemeier and Lemke even took advantage of this fact and constructed a dual mutual orthogonal PylRS OTS for the same stop codon by reprogramming the localization of two PylRS.^135^

Hu et al. relied on a single method, differential scanning fluorimetry (DSF), to determine the stability, which has known limitations when interpreting curves with different shapes, as present in their data.^136^ The temperature differences they observed are too small and not consistent to account for the performance differences among the various PylRS variants. Additionally, it seems unlikely that DSF is a reasonable method to assess the melting temperature for large proteins connected with a very flexible linker, that also differs in size and therefore most likely also in flexibilities between samples. Differences in flexibilities likely explain the dissimilar shapes of their melting curves. Despite the rationale behind the improvements being unclear, they still identified two variants with better performance in mammalian cells.^92^ Another compelling study highlighting the problematic behavior of +N class PylRS with respect to the NLS was conducted in yeast.^137^ There it was demonstrated that the *M. alvus* PylRS, which belongs to the ΔN class, significantly outperforms other +N class PylRSs in yeast. Most of the +N PylRSs showed no activity in their setup. Therefore, to improve the performance for +N class PylRS in eukaryotic cells, careful consideration of the NTD with its associated NLSs is essential, and direct targeting of the NTD for improvements may be necessary.

## 5 Coupling Metabolic Engineering and PylRS-Based OTS

The coevolutionary theory of genetic code evolution suggests that the amino acid repertoire expanded concurrently with the synthetic capacities of evolving cells, progressing from the earliest stages of genetic coding through the hypothetical organism LUCA to contemporary life.^138^ According to Wong, α-amino acids, serving as stable metabolic intermediates, were recruited as building blocks for proteins during the genetic code evolution process.^138^ Despite the code being “frozen” to 20 (+2) building blocks, this evolutionary innovation can be extended to GCE-OTS technology, allowing the “unfreezing” of the genetic code.^139^ Looking forward, the success of GCE will significantly depend on its capacity to integrate ncAAs produced from simple metabolic precursors into the OTS. This integration of metabolic engineering with OTS has the potential to create synthetic cells as robust and programmable production units.^140^

In the context of GCE, while it is an invaluable tool, there are still some limitations for large scale productions. In a conventional GCE-based experiment or production process, the cost of ncAA supply can indeed pose a challenge to the feasibility of a project. To facilitate the adoption of GCE as a general tool in basic and applied sciences and to broaden its application areas, two key criteria must be addressed. The first is easy implementation, which is largely achieved since most PylRS OTS are a one plasmid system and ready to use with simple transformation/transfection or even genomically integrated in prokaryotic^141^ or eukaryotic^142^ cells. Detailed protocols are also abundantly available to guide this implementation if necessary.^143^ The second criterion is the cost of producing the desired target protein, which can still be a significant hurdle. Strategies to reduce costs are essential, and various approaches can be considered.^144^ One approach is to increase the *in vivo* efficiency of the used PylRS OTS, as discussed in section 4. Another solution involves the use of inexpensive ncAAs, if suitable for the desired goal. For example, substrate **303** could be a cost-effective candidate for bioconjugation. Unfortunately, numerous ncAAs currently in use are far more expensive compared to **303** and are expected to stay like this, prompting the exploration of an alternative approach: the synthesis of ncAAs through endogenous pathways utilizing economically cost-effective precursors, ideally in standard cultivation medium.^144^ This entails the introduction of metabolic engineering in the field and its blending with OTS.

**Figure 4:**
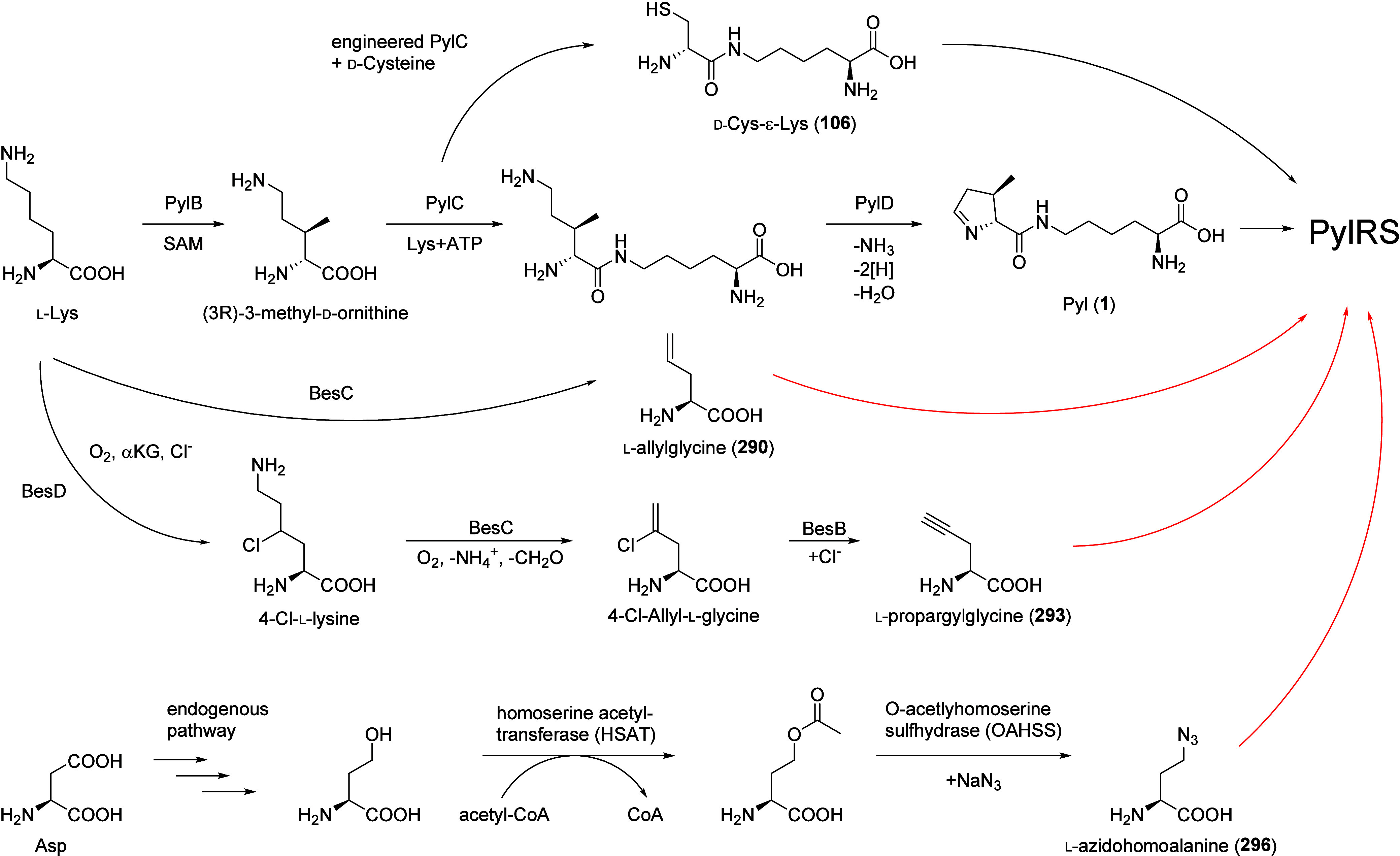
Natural and engineered Pyl-biosynthesis pathways. The natural Pyl biosynthesis^145,148^ was repurposed by establishing a new Pyl route via engineered PylC.^144^ The potential routes for incorporating ncAAs are highlighted in red, using existing biological synthetic pathways^146,147^ in combination with engineered PylRS variants.^26^ The metabolic pathways outlined in red, along with the specific PylRS variants facilitating the incorporation of these ncAAs, have been documented in the literature. The missing link is establishing the connection and optimizing these components to create a complete functional GCE pathway.

The *M. acetivorans pylBCD* pathway was transplant to *E. coli* and engineered to yield a robust and efficient synthetic biological pathway ready for GCE.^145^ Tai and co-workers, for example, repurposed parts of the natural Pyl biosynthesis machinery by engineering PylC to recognize d-cysteine, which is normally not present in any cultivation medium but still very cheap/cost-effective.^144^ This led to a system capable of incorporating d-Cys-ε-Lys (**106**) by only supplying LB medium and d-cysteine to *E. coli*. Three other substrates, **290**, **293**, and **296** (see Figure 4), appear feasible for coupling with the endogenous production of *E. coli*. Marchand and co-workers recently demonstrated that **290** and **293** can be produced *in vivo* in *E. coli*.^146^ Recently, it was demonstrated that **296** can be produced by an engineered cysteine biosynthetic pathway in *E. coli*,^147^ requiring the addition of sodium azide, which is a cheap precursor, to yield **296**. PylRS variants which can incorporate these three substrates were recently reported.^26,27^ To make these systems functional, more efficient PylRS variants have to be to evolved and/or the biosynthetic pathways have to be optimized to yield intracellular ncAA concentrations which are sufficiently high. In-cell ncAA concentrations of 50–100 μM were reported, which is the lower end of what very efficient PylRS enzymes can currently work with.^27^

## 6 Applications of Noncanonical Amino Acids

**Figure 5:**
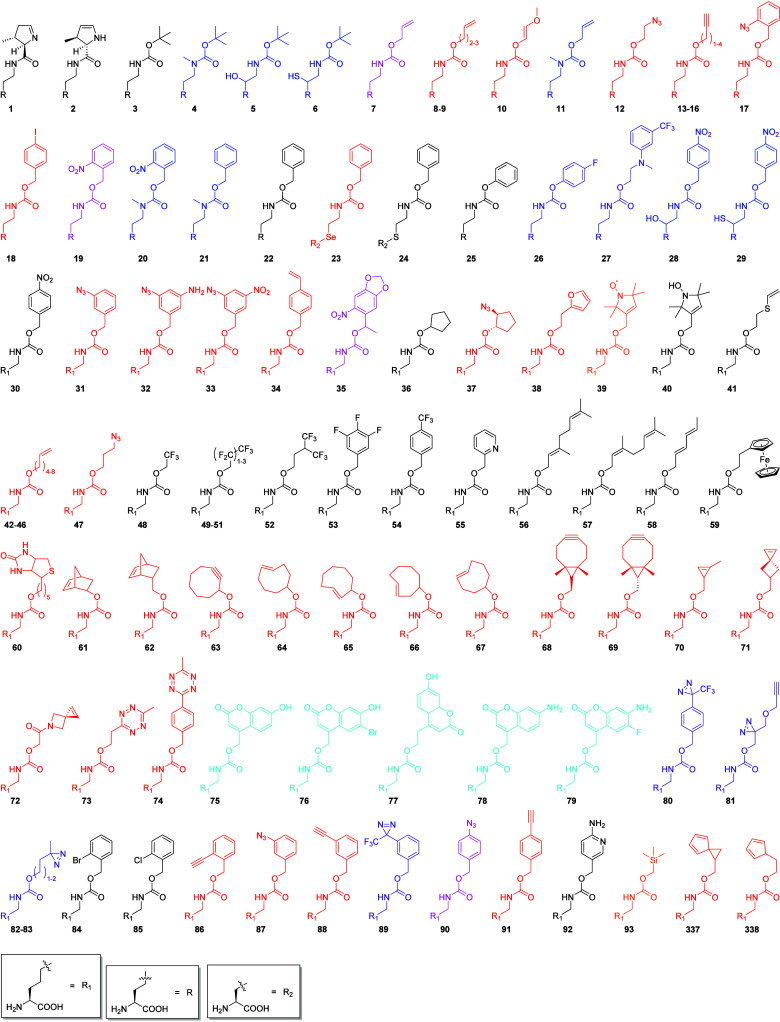
Group 1 of Lys derivatives that can be incorporated *in vivo* using the PylRS OTS. All ncAAs are Lys-derivatives containing a carbamate group starting at the Lys N^ε^. Most of these ncAAs can be incorporated with the wild-type or PylRS(Y306A:Y384F, *M. mazei* notation) variant. Photo- or chemically caged post-translational modifications (blue), ncAAs containing bioorthogonal groups and are possible targets for site-specific bioconjugation (red), photo- or chemically caged ncAAs or cAAs (magenta), ncAAs containing functional groups which are useful for spectroscopic applications (orange), photo- or proximity triggered cross-linking ncAAs (violet), fluorescent ncAAs (cyan).

**Figure 6:**
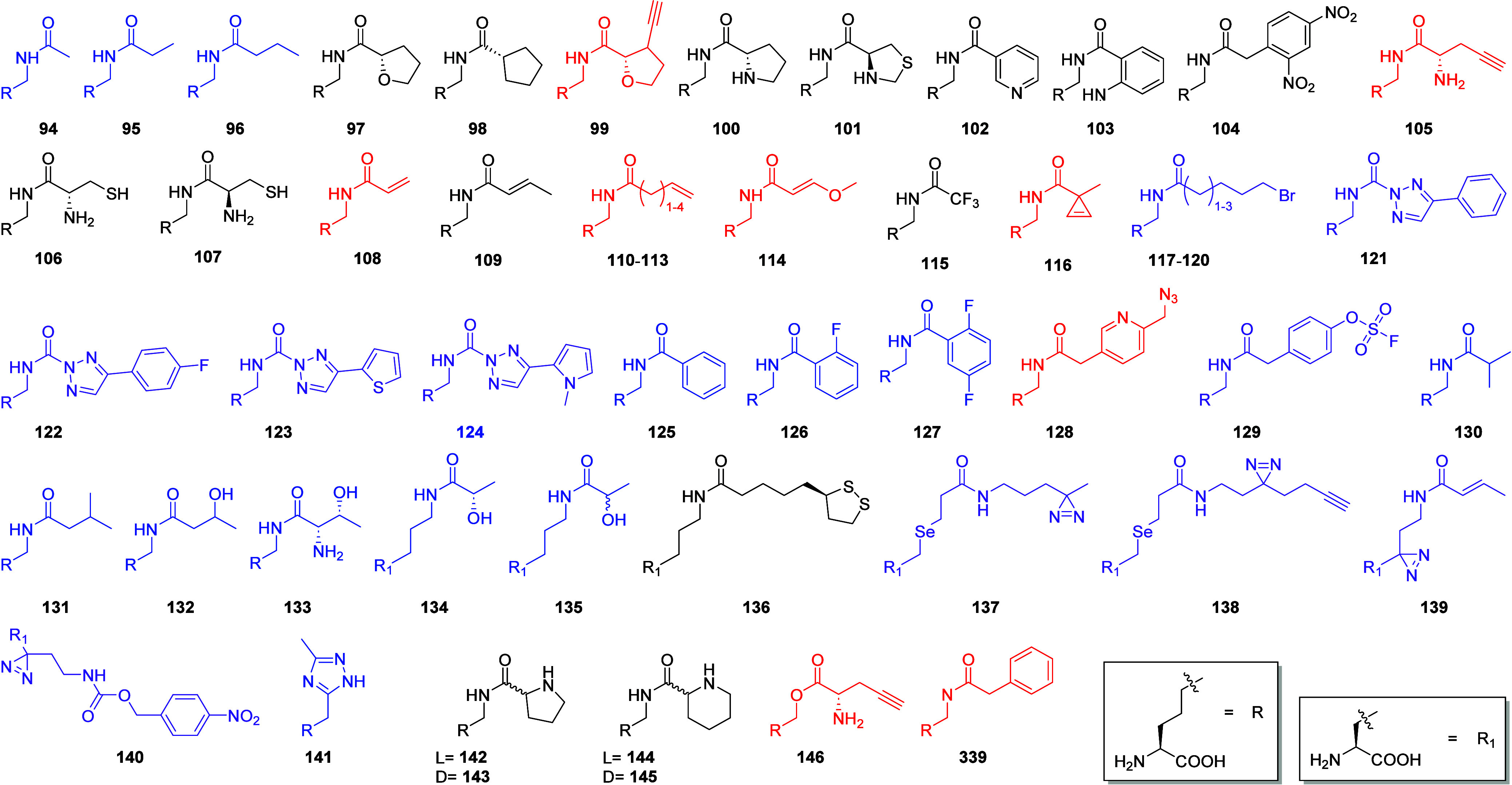
Group 2 of Lys derivatives that can be incorporated *in vivo* using the PylRS OTS. All ncAAs are Lys-derivatives with a Lys N^ε^ amide, or one with and ester (**146**). **140** and **141** are exceptions due to space constrains. Caged or noncaged post-translational modifications (blue), ncAAs containing bioorthogonal groups and are possible targets for site-specific bioconjugation (red), photo- or proximity triggered cross-linkable ncAAs (violet).

**Figure 7:**
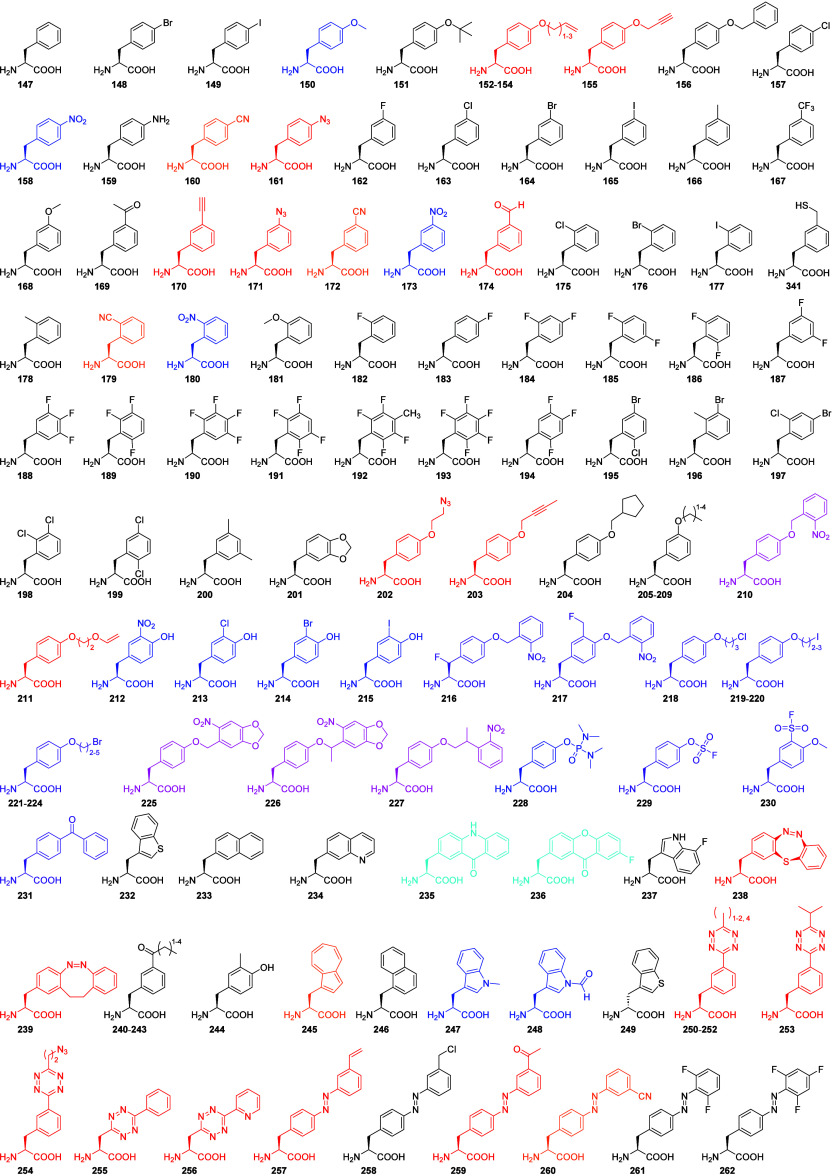
Group 3 contains short and bulky or larger bulky non-Lys ncAAs that can be incorporated *in vivo* using the PylRS OTS. Post-translational modifications (blue), ncAAs containing bioorthogonal groups and are possible targets for site-specific bioconjugation (red), photocaged ncAAs or cAAs (magenta), ncAAs containing functional groups which are useful for spectroscopic applications (orange), photo- or proximity triggered cross-linkable ncAAs (violet), fluorescent ncAAs (cyan).

**Figure 8:**
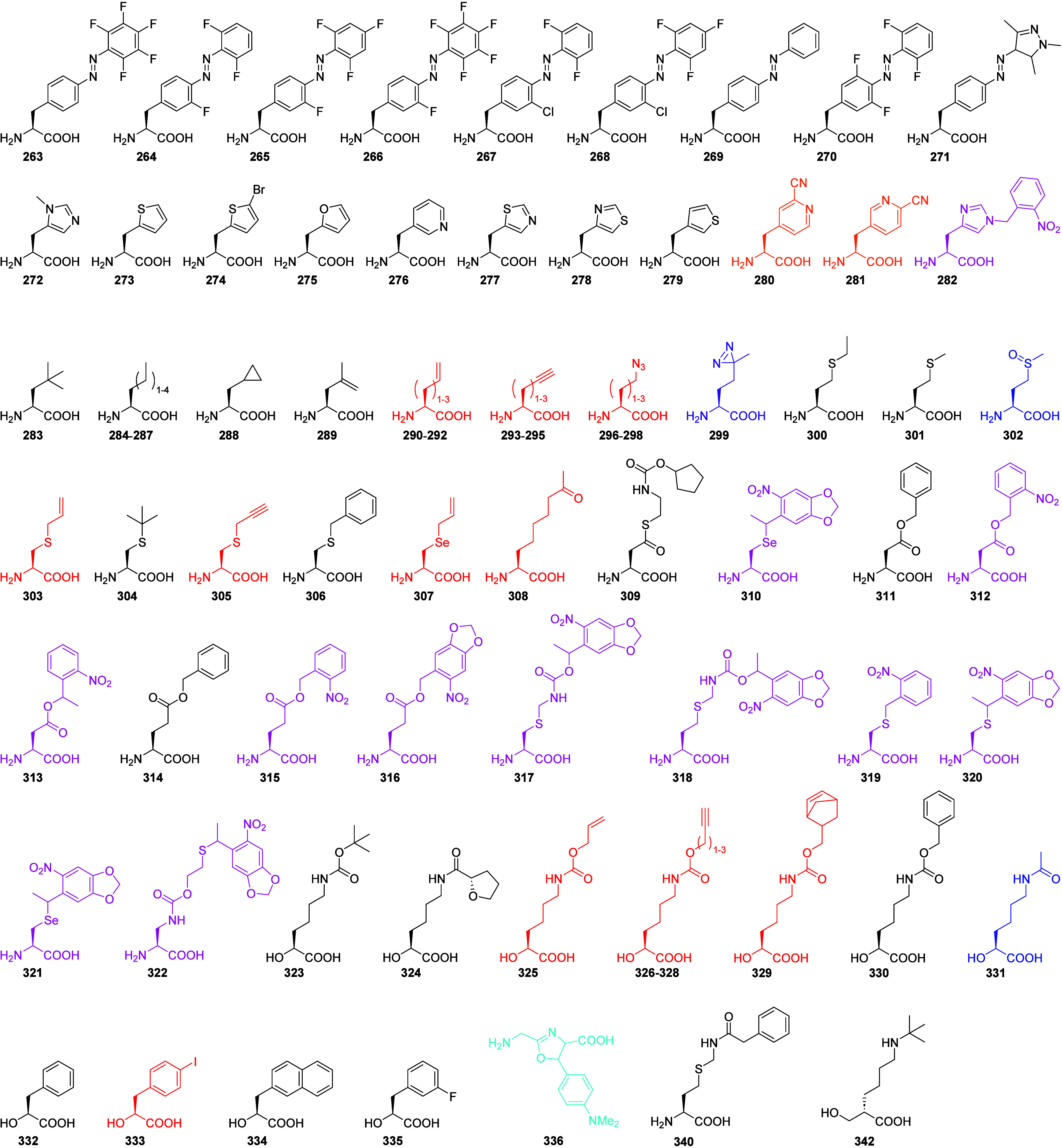
Group 4 contains small and bulky His analogs, small aliphatic and small caged ncAAs. This group also contains the unusual α- and β-hydroxy acids, and all of them can be incorporated *in vivo* using the PylRS OTS. Some of the Lys derivatives contain ester which are not that common in the GCE field. Also, the *in vivo* incorporable α-hydroxy acids are very unusual substrates. They especially highlight the unique substrate promiscuity of the PylRS OTS. Post-translational modifications (blue), ncAAs containing bioorthogonal groups and are possible targets for site-specific bioconjugation (red), photocaged ncAAs or cAAs (magenta), ncAAs containing functional groups which are useful for spectroscopic applications (orange), photo- or proximity triggered cross-linkable ncAAs (violet), fluorescent ncAAs (cyan).

GCE provides a platform for reprogramming of cellular functions across multiple levels, showcasing a versatility rarely observed in molecular biology and biotechnology. The examples presented in this section demonstrate GCE as an enabling technology, employed in diverse ways that defy clear-cut classifications due to their innovative nature. We have categorized these applications into four groups, with three sharing a common core theme, while the fourth serves as an outgroup, encompassing promising concepts that may not necessarily share direct conceptual connections. Also, we assembled the ncAAs into distinct groups as depicted in Figures 7 and 8, roughly based on their chemical similarity and size. Additionally, for Lys-derivatives, we categorized them according to the connection of functional groups attached to the Lys side chain, such as carbamates (Figure 5), amines, esters, and carbamides (Figure 6), with a single exception due to space constrains (**140**). This grouping was adopted to assist experienced GCE practitioners in identifying specific enzyme regions that most likely underwent mutation, or to ascertain if they possess PylRS variants in their laboratory capable of incorporating ncAAs they were not aware of. Importantly, the color coding serves to emphasize a particular application, either as used in the original publication or the one we deemed most significant by our assessment. Of course, for several ncAAs, there exists more than one application. For further details, please use the supplemented ncAA table.

### 6.1 Probing and Engineering Protein Structure and Function

Protein engineering can be broadly categorized into two classes. The first is reverse engineering, where a chosen protein is modified to gain insights into the native biological mechanism. The second class is forward engineering, involving the alteration of a protein’s function, either to enhance its native function or to confer entirely new functions not observed previously.

#### 6.1.1 Probing Proteins for Bioanalytics (Reverse Engineering)

There is a plethora of traditional bioanalytics approaches; however, the question arises: why opt for GCE to gain information about a protein or a cellular biological question? Typically, two approaches are employed to elucidate specific aspects of natural phenomena. The first involves observing/analyzing the undisturbed/natural system of interest. Given the intrinsic complexity of biological systems, including proteins, the mere observation of these systems often yields limited insights. The second, more prevalent approach in molecular biology, is to perturb the system, analyze the resulting changes or recovery behavior, or place the target part to be analyzed into an artificial environment for more detailed study.

While this second approach provides researchers with far more degrees of freedom to manipulate and analyze a target system, it raises the critical question of whether the analyzed system resembles the native state close enough or if an artificial system is being measured, especially in the context of complex biological systems. This trade-off of acquiring less detailed but biological relevant data and acquiring very detailed data while measuring nonrelevant biological artifacts is a common challenge in the life sciences. This dilemma is often referred to as the *in vitro*–*in vivo* problem and underscores the difficulty of mapping a specific aspect of a system in question in a more complex environment. To address this issue, it is always desirable to obtain data of an undisturbed or minimally disturbed biological target system.

The incorporation of ncAAs into proteins enables alterations that can be tailored in terms of desired physicochemical properties and magnitude of disturbance, ranging from the smallest possible alterations (such as atomic mutations^149^) to very large ones. ncAAs equipped with specific chemical groups (azides, olefins, ketones, and aldehydes, alkynes, halogens, oximes, hydrazones, boronic acid esters and acids, etc.) confer unique reactivity and chemoselectivity to polypeptide sequences. This enables easy and efficient bioconjugations to a variety of ligands. Importantly, the incorporation of ncAAs even enables the study of protein features in native biological environments, a capacity that was not possible with traditional methods. When executed correctly, these data are significantly more biologically relevant compared to results obtained through more traditional *in vitro* approaches.

As a quick summary, GCE can be used to introduce spectroscopic probes (vibrational, IR, EPR, NMR, fluorescent, FRET),^150−156^ redox and p*K*a probes,^157−159^ selective reactive groups,^160,161^ PTMs,^17,162^ cross-linker,^11^ elucidating enzyme substrates by trapping,^21^ photo- and chemically caged amino acids,^163−167^ and also combinations of these functions, e.g., photo controlled bioconjugation/cross-linking/trapping.^168−170^ At a cellular level, GCE can be used to elucidate protein localization and interactions using methods like microscopy^132,171−173^ or mass-spectroscopy-based proteomics.^28,174^ A notable development in structural biology involves the use of GCE, combined with mass-spectroscopy-based proteomics, to improve protein structure predictions generated by AlphaFold2.^175^

#### 6.1.2 Engineering New-to-Nature Protein Properties and Functions (Forward Engineering)

**Figure 9:**
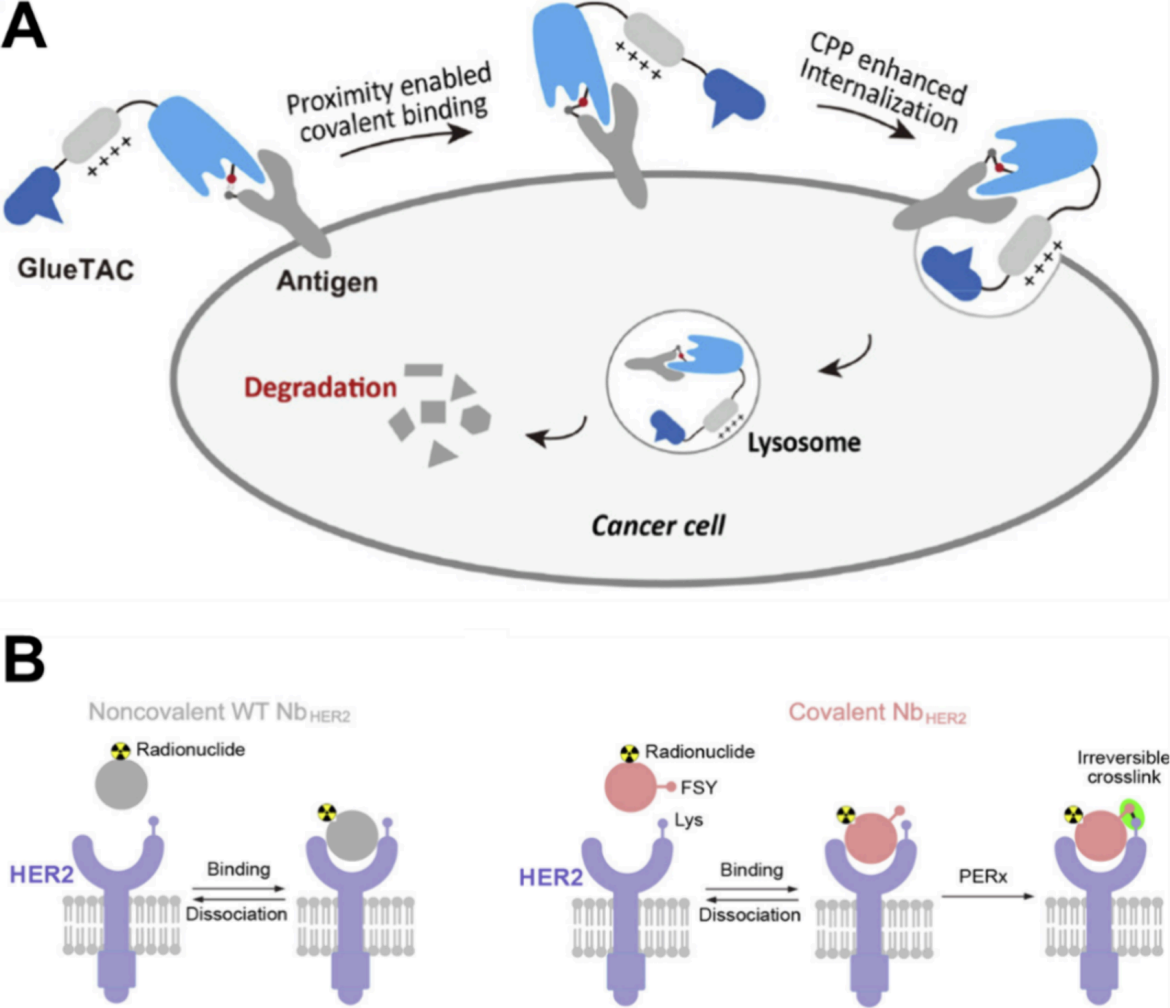
A) Nanobody containing **229**, with a cell-penetrating peptide (CPP) tail, targeting PD-L1. B) Schematic comparison of the binding mechanism for conventional a nanobody and a covalently binding nanobody containing FSY(**229**), both targeting the HER2 receptor. Adapted from refs (226, 227). Copyright 2021, 2023 American Chemical Society.

An important aspect of GCE is to confer proteins with functions not observed in nature. We want to emphasize that some of the functionalities mentioned in this paragraph overlap with the concept of reverse engineering from the section before. For instance, proximity reactive groups can be used for covalent cross-linking of target binding scaffolds like anti- or nanobodies (Figure 9).^160,161^ This strategy enhances target affinity, as covalent bonds have no off-rate, providing “infinite affinity”. This feature is particularly useful since binding scaffolds are typically optimized for their specificity and binding affinity. By encoding an ncAA with a proximity reactive group, only specificity needs to be optimized. Another example that falls in both categories is the use of photocaged ncAAs which can be employed to create new molecular functionalities that are turned on (activated) upon exposure to light.

A significant goal in protein engineering is achieving fully programmable protein catalysis.^176−178^ In this context, the incorporation of ncAAs into enzymes also plays a key role to enable these enzymes with new-to-nature functions.^179^ New-to-nature functionalities that have already been realized include the following. In organic synthetic chemistry, particularly organocatalysis, the use of small molecules as catalyst is a standard approach with 4-(dimethylamino)pyridine (DMAP) being one of the oldest and most versatile catalysts.^180^ Recently, this mode of reactivity was transferred to a hydrolase by replacing a His residue with 3-methylhistidine (**272**) creating an enzyme with a mode of action not observed before.^181^ Interestingly, there are enzymes that naturally contain methylated His residues known as lytic polysaccharide monooxygenases (LPMOs).^182,183^ The function of the methylation is thought to protect the His residue from oxidative damage when H_2_O_2_ concentrations are too high. It would be intriguing to explore if similar properties could be transferred to other enzymes. Given the industrial significance of LPMOs, the ability to encode **272** without relying on post-translationally modifying enzymes is now possible and could be advantageous.

For the 2021 Nobel Prize awarded asymmetric organocatalysis, the usage of chiral amines, e.g., proline, is common.^184,185^ In biology, the involvement of proline in a catalytic mechanism is extremely rare since proline is normally a structural breaker due to its restricted backbone dihedral angles. Recently, two proline-analogous ncAAs have been incorporated into proteins to transfer the reaction properties of asymmetric organocatalysis into the world of enzyme catalysis.^186^

In this context, PylRS-based OTS holds great potential, as Pyl itself can be viewed as a composite molecule comprising a methylated/oxidized proline analog and lysine modules (in fact, 4-methyl-pyrroline-5-carboxylate ring attached to the lysine side chain). Recently, a series of Pyl derivatives with d-proline and analogs have been generated and incorporated into proteins, conferring on them unique catalytic properties.^187^ Both Pro and Pyl are not only the oldest amino acids in the amino acid repertoire of the genetic code but also important to produce homochiral sugars and mediation of chemical transformations that were vital in the early stages of evolution (such as transfer hydrogenations or transaminations).^188^

The capability to incorporate ncAAs have proven beneficial in various fields, including the development of artificial metalloenzymes^189^ to coordinate the right metal-ion or the creation of artificial enzymes in terms of new substrate recognition.^190^ It is intriguing to observe how advancements in biotechnology and synthetic biology have facilitated the transfer of ideas and concepts initially applied only in synthetic organic chemistry to enzyme catalysis and it is expected that additional ones will follow.

Although thermal stabilization of enzymes cannot be considered a new-to-nature functionality, it is worth noting that this can also be achieved through the use of ncAAs.^191^

Proteins and enzymes are not the only molecules where GCE can be applied. Another significant class involves biomaterials (protein-based polymers) with the goal of using these materials for example for tissue engineering or drug delivery.^19,20,192^ One of the key advantages in using protein-based polymers is the ability to precisely control the length and composition of these polymers through genetic encoding. This level of precision is challenging to achieve in chemical polymer synthesis. Furthermore, with the incorporation of ncAAs, these biomaterials can be endowed with a variety of new-to-nature functions. Examples include introducing photocontrol of a critical phase transition temperature^193^ or enabling precise drug conjugation.^20^

### 6.2 Site-Specific Protein Conjugation

Undoubtedly, one of the main drivers in developing and expanding GCE technology has been the pursuit of achieving site-specific protein modifications with reactive handles or bio-orthogonal tags. In this context, we aim to provide a concise overview of the historically important, widely used, and versatile approaches, as well as methods with unexplored potential. For the reactions discussed, we will provide examples of ncAAs that can be used, where applicable. It is important to emphasize that this overview is not exhaustive, as comprehensive coverage exceeds the scope of this Review. For readers interested in a general overview of protein conjugation strategies, we refer to the excellent review by Boutureira and Bernardes.^194^ Additionally, for insights into mutual orthogonal bioorthogonal chemistry, the work of Smeenk and colleagues is recommended.^195^

#### 6.2.1 Oxime Ligation

Although not extensively studied in a biological context, oxime formation reactions have been a subject of research since as early as 1882, making it one of the oldest bioconjugation reactions.^196^ While not strictly bioorthogonal, the rare occurrence of carbonyls and aldehydes in biological systems has made it possible to selectively target these specific functional groups with notable success for many applications.^196^ When incorporated into recombinant proteins, aldehydes (**174**) and carbonyls (**169**, **308**) can be specifically addressed for oxime ligations.^197,198^ Unfortunately, the requirement for harsh reaction conditions (pH = 4–5) and the slow reaction kinetics (ranging from 10^–4^ to 10^–3^ M^–1^ s^–1^) impede their universal applicability. Extended incubation times, typically ranging from 1 to 4 days, are needed for high bioconjugation yields.^199,200^ Despite these limitations, this chemistry is employed in the development of at least one antibody–drug-conjugate set to enter the market in the near future (note this is not done with the PylRS system).^201,202^

#### 6.2.2 Staudinger Ligation

The Staudinger ligation, the first reported completely bioorthogonal reaction, was pioneered by Saxon and Bertozzi.^203^ This ligation takes advantage of the selective reactivity of phosphines and azides, resulting in the formation of an amide bond. The first application of this reaction involving ncAAs was done with a proteome-wide sense codon reassignment of the Met codon, using Azidohomoalanine (**296**) with consecutive conjugation.^204^ The intracellular metabolic biosynthesis of **296** and its use for protein translation (as a substitute for Met) is also well documented.^205,206^ With the successful incorporation of **296** with the PylRS system, this ncAA was transitioned from auxotrophy-based^207^ insertion to site-specific insertion. Now, proteins can be endowed with a terminal azide, providing site-specific bioorthogonal handle/tag.^26^ In general, many ncAAs discussed in this Review (Figures 5–8) contain an azide and are therefore potential candidates for this type of ligation.

#### 6.2.3 Copper(I)-Catalyzed Alkyne–Azide Cycloaddition (CuAAC)

The copper(I)-catalyzed alkyne–azide cycloaddition (CuAAC) is the first example of a bioorthogonal “click chemistry” reaction reported by Finn, Sharpless, and co-workers.^208^ An azide and alkyne led to the formation of stable 1,4-triazole conjugates in the presence of a Cu(I) catalyst. Unfortunately, this reaction suffers from the side-product formation of reactive oxygen species induced by the use of copper, ascorbate and atmospheric oxygen. This issue could be reduced by designing new Cu(I) catalysts and optimizing the ascorbate concentrations.^209,210^ The latest improvement of this reaction, in terms of reducing Cu(I) toxicity and accelerating reaction kinetics, came from Uttamapinant and co-workers by using picolyl azides, which Meineke and colleagues translated to **128** for use in GCE.^211,212^

#### 6.2.4 Strain-Promoted Alkyne–Azide Cycloaddition (SPAAC)

To circumvent the drawbacks of CuAAC but still have a click reaction, Bertozzi and co-workers reported the strain-promoted alkyne–azide cycloaddition (SPAAC).^213^ In SPAAC, the adaption in comparison to the CuAAC is that the alkyne is under ring strain which triggers the reaction, no catalyst needed. Often moieties containing a cyclooctyne ring are used. Unfortunately, the reaction kinetics are slower than CuAAC, but this can be improved by using cyclooctyne variants containing a difluormethyl group in proximity to the alkyne bond or using electron deficient azides (**33**).^214,215^ Aza-dibenzocyclooctyne (DBCO) was developed to further increase favorable kinetics and hydrophilicity.^216^ Additionally, the orthogonality of this molecule with the tetrazine-based IEDDA reaction (see below)^195^ was responsible for an almost exclusive use of this alkyne group in SPAAC reactions, at least if one counts commercial availability as an indicator. However, as a result of the designed increased reactivity, the bioorthogonality of this method has some drawbacks with observed side reactions of the cyclooctyne moieties with cellular and plasma nucleophiles (e.g., the sulfhydryl side chain of free Cys) are observed.^217^ As mentioned above, a substantial amount of ncAAs contain azides and alkynes from all classes, most of them tested for SPAAC or CuAAC.

#### 6.2.5 Inverse Electron Demand Diels–Alder (IEDDA) Reaction

The tetrazine and alkene-based inverse electron demand Diels–Alder (IEDDA) reaction, pioneered by Devaraj, Weissleder, and Hilderbrand, is a class of reaction with the highest degree of bioorthogonal properties.^218,219^ Since the IEDAA finds extensive applications beyond the realm of GCE, a comprehensive overview is provided by Oliveira and Bernardes.^219^ While numerous ncAAs can participate in this reaction, substrate **303** distinguishes itself due to its superior incorporation efficiency, stability under biological conditions, and affordability. The cost of **303** is so low that it is possible to install this moiety into the target proteins without incurring additional costs compared to the wild-type protein.^27^

#### 6.2.6 Transition-Metal Catalyzed Reactions

In addition to the Cu-based CuAAC and SPAAC, there are a few other transition-metal catalyzed reactions which can be performed with several ncAAs. Palladium-based Suzuki-Miyaura, Heck, and Sonogashira reactions are among them, as well as Ru-based olefin cross-metathesis. A comprehensive description can be found in the review from Boutureira and Bernardes.^220^ Here, we emphasize the significance of substrate **303** for Ru-based cross-metathesis, owing to its enhanced reactivity of allyl sulfides over other allyl compounds and its facile and efficient incorporation into target proteins.^27,221,222^

#### 6.2.7 Thia-Michael Addition

Similar to the oxime ligation, the thia-Michael addition is not strictly bioorthogonal since the nucleophile in this reaction is a thiol/thiolate. Leveraging the nucleophilic properties of cysteine is a classical and widely adopted approach, given that cysteine is a relatively rare amino acid, with only a fraction being surface exposed, thereby minimizing off-target reactions.^194^ Normally a surface exposed site of a target protein will be mutated to a Cys residue which can then be targeted. Since cysteines are rare but still present in most proteins, this is a major drawback.^223^ Additionally, the thiosuccinimide linkage formed in traditional strategies is instable under physiological conditions, severely limiting the applicability of this approach.^224^ These disadvantages can be circumvented by inverting the directionality of this conjugation strategy, incorporating ncAAs **108** or **109**, for example, as an ncAA with a Michael acceptor side chain that can then be conjugated with an appropriate thiol in a thia-Michael addition.

### 6.3 Therapeutic Applications

#### 6.3.1 Targeted Therapies

Li, Chen, and Klauser recently demonstrated the transfer of the concept of small molecule covalent drugs to protein drugs, creating a novel drug class.^225^ By incorporating ncAA **229** into human programmed cell death protein-1 (PD-1), they introduced a proximity-triggered cross-linker that reacts with specific nucleophiles of cAA side chains. This property allowed for the irreversible binding of PD-1 to only PD-L1. Administrated to immune-humanized mice, the covalently bound PD-1 exhibited a more significant antitumor effect than the noncovalent wild-type PD-1.^225^ A similar strategy was employed by introducing ncAAs **229** and **120** into a nanobody which also targeted PD-L1^226^ or human epidermal growth factor receptor 2 (HER2),^227^ shown in Figure 9. This nanobody demonstrated high efficiency in degrading PD-L1, leading to sustained T-cell activation, tumor growth inhibition, and superior results compared to Atezolizumab.^226^ Further development of covalent binding concept resulted in a light-activatable covalent antibody fragment (7D12).^228^ It was demonstrated that upon light irradiation, their antibody fragment covalently binds to the epidermal growth factor receptor target achieved by introducing ncAAs **210** and **231** into 7D12. In general, the innovative approach of covalent protein drugs holds promise for enhancing their effectiveness and specificity.

Even though the first review about antibody–drug conjugates (ADCs) by Ghose and Blair was published in 1978,^229^ they represent a relatively new class of therapeutics with Gemtuzumab-Ozogamicin (Mylotarg) being the first FDA-approved ADC for human use in 2000.^230^ ADCs consist of an antibody determining the biological target and are coupled with a toxic molecule (payload) that would be too toxic for a global mode of action if administered independently. Early ADCs were conjugated at Lys side chains, resulting in significant heterogeneity in the product. Despite improvements in antibody conjugation chemistries,^231^ the challenges associated with synthetic chemistry, particularly due to the sheer size of antibodies and the corresponding number of cAAs, remain a persistent issue.

In contrast, one of the most common drawbacks unrelated to the antibody size is associated with current conjugation chemistry, which can lead to the premature release of the toxic payload, causing off-target toxicity and decreasing the therapeutic window.^232,233^ Here, GCE appears to be a straightforward solution to address both the heterogeneous product-formation and off-target toxicity issues, offering the potential to design ADCs with new modalities (improved properties). GCE has indeed emerged as a technology for creating ADCs with enhanced characteristics, introducing novel possibilities with examples such as ncAAs **12**,^234,235^**338**,^236,237^**337**,^238^**70**,^239^**13**, **63**, **65**, and **68** (all^240^).

**Figure 10:**
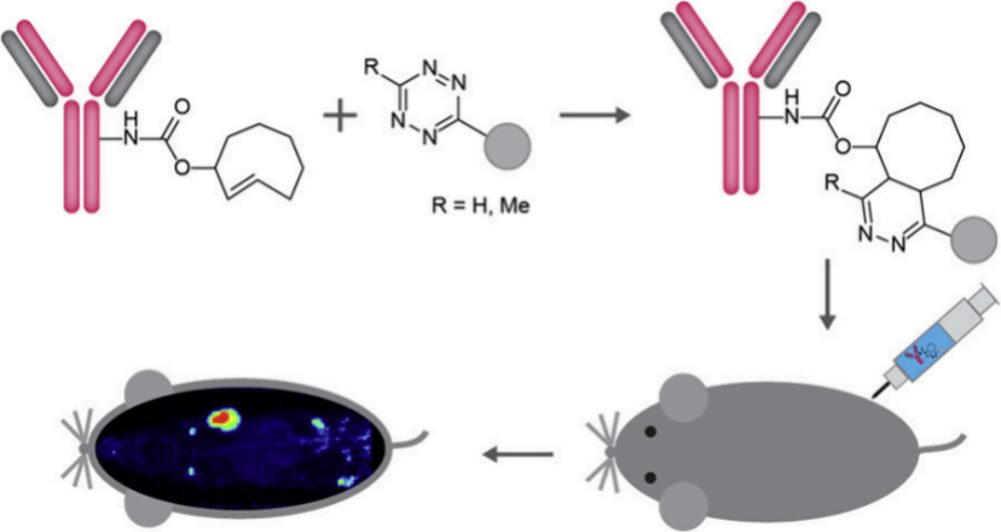
Depiction of the site-specific conjugation of antibodies with radioisotopes for diagnostics and/or therapeutic applications.^244^

The concept of using an antibody or nonantibody binding scaffold to direct a payload to a specific target can be mixed and matched. For example, the payload has been changed to other functional molecules, e.g., oligonucleotides,^241^ enzymes (proteases) for targeted degradation of cancer-associated mucins^242,243^ or the conversion of prodrugs,^243^ and radioisotopes which enable diagnostic and/or closely related therapeutic applications (Figure 10).^244^ Also the assembly of nanobodies into polyvalent multimers increasing the accessible range of nanobody assembly topologies was performed using ncAA **250**.^245^ This shows that GCE is well suited to create protein complexes that bind two or more targets in a mix-and-match approach.

**Figure 11:**
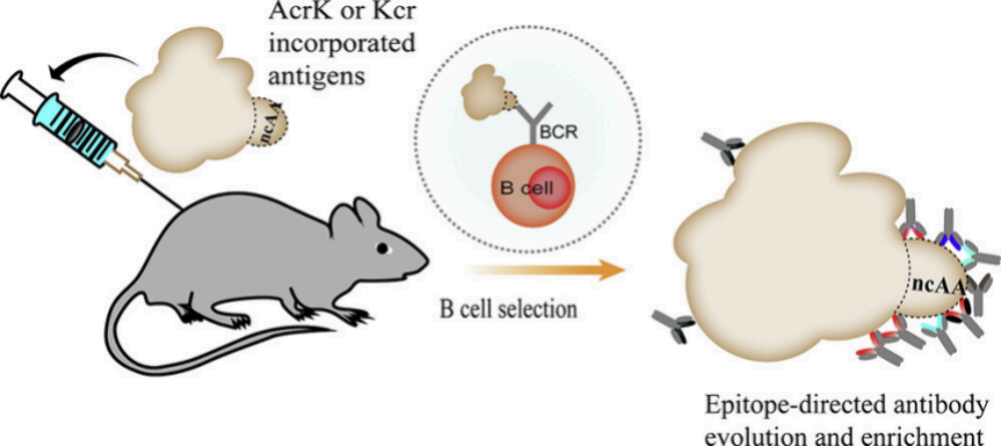
Schematic depiction of epitope-directed antibody elicitation in mice with AcrK (**108**) and Kcr (**109**).^251^

While the concept of ADCs was revolutionary^9^ to increase the concentrations of drugs in target locations otherwise not possible, several limitations still exist. These are intertwined with the use of antibodies and/or current conjugation strategies, including off target toxicities,^246^ relatively low drug- antibody ratios,^246^ and poor solid tumor penetration.^247^ Some of these drawbacks are mainly size correlated, with antibodies having a typical size of around 10 nm. These limitations have led to an ongoing search for alternative targeted nanodelivery vehicles.^248,249^ A considerable improvement on this front came from Zhang and colleagues by creating ultrasmall anti-HER2 drug-immune-conjugate nanoparticles with a size of less than 8 nm.^250^ This was achieved by using multivalent fluorescent core–shell silica nanoparticles that carry multiple anti-HER2 single-chain variable fragments (scFv) and drug molecules. They used GCE to incorporate ncAA **12** into the scFv, which could then be attached to the nanoparticles in a defined orientation. The advantage is that if this drug misses the target, the small size leads to fast renal clearance, lowering the probability of off target toxicities. This translated into a therapeutic index with a wide margin of safety.

#### 6.3.2 Immunotherapeutic and Vaccine Approaches

Spectacular results have emerged from the work of Zhu, Xu, and co-workers.^251^ They used **108** and **109** to elicit epitope-directed antibody responses in mice (Figure 11). **108** and **109** are both Michael acceptors and can react with amines (Lys) or thiols (Cys) to form covalent bonds. The mode of action is similar to the covalently binding scaffolds drug class from the chapter before, with the difference that the “target” in this approach are most likely B-cell receptors. They harnessed the proximity inducible cross-linking properties of **108** and **109** to elicit a specific immune response depending on the epitope where the ncAAs were incorporated. B-cell receptors were covalently bound to specific positions, leading to affinity maturation and resulting in high titers of antibodies binding the exact epitope.^251^ This innovative approach could potentially revolutionize the design of protein subunit vaccines, as it offers a way to induce an antibody response for specifically chosen epitopes. Further studies are required to determine the binding strength of the antibodies generated using ncAAs **108** and **109** and to assess whether this technique can generally produce neutralizing antibodies for epitopes of scientists choosing.

Another promising study uses engineered lung cancer vaccines using live but nonreproductive influenza A viruses with chimeric antigenic peptides.^252^ In this study, **12** neoantigens were fused to surface proteins of these viruses. When administered to the lung of mice, these vaccines triggered a robust immune response that resulted in increased tumor-specific cytotoxic T cell infiltration into the tumor.^252^ Doubtless, this has significant potential for advancing cancer immunotherapy.

In biology, information about a systems behavior is often encoded by protein–protein interactions. By blocking certain sites of a protein, certain pathways can be switched off but retain others that are important. This was achieved by reprogramming of the human cytokine interleukin 2 (IL-2) resulting in THOR-707.^253^ Through site-specific incorporation of ncAA **12** and subsequent PEGylation, THOR-707 selectively engages the IL-2 receptor beta/gamma complex without engagement of the IL-2 receptor alpha. This targeted approach enhances drug accumulation in the tumor tissue, stimulated tumor-infiltrating CD8^+^ T and NK cells, and resulted in a dose-dependent reduction of tumor growth.^253^ This innovative strategy holds promise for improving the efficacy and specificity of cancer therapeutics.

#### 6.3.3 Antimicrobials

There is an ongoing crisis of antimicrobial resistance.^254,255^ For some infections where there used to be an antibiotic treatment, there is already no functional drug available.^256^ In the last 40 years, roughly 60% of all new antibiotics or at least the lead structures were derived from natural products (NPs).^257^ These antimicrobial peptides (AMPs) typically consist of a variety of ncAAs, nonalpha and/or d-amino acids which are well in the scope of GCE.^258,259^ Unfortunately, traditional natural product discovery methods do not scale well making the process slow and having a low success rate.^260^ While there are some advancements in *in silico* methods to identify the gene clusters responsible for the production of AMPs, the entire process remains slow and challenging.^258^

Therefore, we envision that in the not-so-distant future, a genetically encoded AMP screening workflow, where AMPs contain multiple ncAAs, can be established with the help of the GCE technology.^261^ Initial experiments aimed at expanding the chemical space of AMPs using ncAAs^262^ were conducted in the frame of auxotrophy-based approaches,^263,264^ whereas among the first GCE experiments were those based on *Mj*TyrRS-OTS using nisin A^265^ as a model lantibiotic with a few examples of combined approaches.^266^ Regarding PylRS-based OTS, a promising start for this endeavor has been undertaken by Robertson, Funke, de la Torre, Fredens, and co-workers. They generated peptide repeats with different combinations of two ncAAs (**3**, **7**, **22**, **149**).^115^ An additional prominent feature of AMPs and their derived drugs is that they are often macrocyclic and frequently contain α-hydroxy acids, resulting in depsipeptides.^258,259,267,268^

In general, cyclic peptides (also often referred to as macrocyclic peptides) are an important class of therapeutics with steady growth in importance. For example, one of the newest antibiotic classes against *A. baumannii* is a cyclic peptide.^269^ In general, cyclic peptides have high binding affinities, low metabolic toxicities, and good stability with some of them even easy to administrate orally.^267,270−272^ In a proof-of-concept study, it was shown that macrocyclic peptides could be produced with the help of GCE.^99^ In this study, the authors could even engineer PylRS variants that can discriminate between ncAAs and α-hydroxy acids. Additionally, two other groups established macrocyclization strategies based on different chemistries.^273,274^ GCE, in general, can enable the *in vivo* production of peptides with functional groups that would be otherwise just impossible to produce by conventional recombinant means. These peptides offer the potential for macrocyclization strategies that would otherwise be impractical.^275^ If these principles can be enhanced to create robust platforms, these could even be coupled with directed evolution to help improve the lead optimization phase and, in turn, boost the early stages of antibiotics discovery, which is currently the main bottleneck.^260^ Achieving these goals hinges on the ability to liberate even more codons from *E. coli* or other organisms beyond the three which have already been freed up,^115^ or if strategies such as quadruplet codons can be established robustly.^276,277^ For more extensive discussion see section 7.2.

#### 6.3.4 Premature Termination Codon

Shi, Yang, and Zhang demonstrated in an impressive *in vivo* study that the restoration of muscle function was possible in two mouse models by readthrough of a nonsense mutation in the dystrophin gene using **12**.^278^ A premature termination codon (PTC) in this gene typically causes Duchenne muscular dystrophy.^278^ Approximately 11% of monogenic diseases involve nonsense mutations that are caused by (PTCs). Based on this study, the use of the PylRS OTS appears to be a realistic option for the therapy of PTC-mediated human monogenic diseases and will hopefully be explored further.^278^

### 6.4 Other Biotechnological Applications

As mentioned above, (cyclic) AMPs are an important focus in drug development. Phage display, a commonly used tool in this context, allows for the exploration of new structures and the improvement of hit compounds.^268^ Oller-Salvia and Chin extended the application of PylRS OTS to phage display campaigns, introducing ncAA **70** into single-chain variable fragments (scFv) displayed on phages.^279^ This approach demonstrates the potential of incorporating ncAAs into displayed peptides to significantly enhance the binding affinity showcasing the versatility of PylRS OTS in various applications.^280^

Increasing affinity is a crucial aspect of various areas, including drug discovery for specific targets. In this context, the vastness of chemical space poses a significant challenge, even with the use of high-throughput chemical libraries.^281^ Tethering is a breakthrough approach where fragments of potential drug candidates are fused to moieties containing a thiol groups. This enables the formation of covalent bonds in proximity to the potential fragment binding site, enhancing their affinity. Tethering has proven to be a powerful strategy in drug discovery, significantly reducing the library sizes needed by improving signal-to-noise ratios. Additionally, covalent attachment in tethering enhances the signal, allowing the use of lower fragment moiety concentrations.^281^

**Figure 12:**
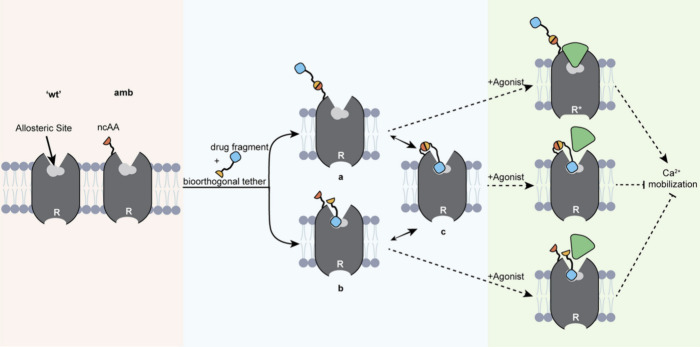
Workflow for pharmacological testing of tetherable heterobifunctional ligands for GPCRs containing a ncAA, adapted from ref (284). Copyright 2023 American Chemical Society.

However, a major drawback of traditional tethering methods is their lack of bioorthogonality, limiting their application to proteins that have only few to no cysteine residues and requiring reducing conditions. The introduction of bioorthogonal tethering using the PylRS OTS, has addressed these limitations and advanced the strategy, expanding its applicability to a broader range of proteins.^282,283^ Mattheisen and colleagues could even target allosteric binding sites by using ncAA **70**, particularly in combination with inverse electron demand Diels–Alder reaction (Figure 12).^284^ They managed to identify high affinity fragments for highly dynamic allosteric sites of G protein-coupled receptors (GPCRs) which were not targetable before. This showcases the scope expansion of structural protein elements that can now be targeted with this method.

We envision the potential for a broader application of biorthogonal tethering concepts to all sorts of molecules, beyond small molecule drug fragments. The logical next step could involve applying this concept to genetically encoded cyclic peptides or producing macrocycle–drug conjugates where the macrocycles act as target binding scaffolds.^272^ These advancements should drastically reduce the ratio of proteins that are not targetable with small molecules nowadays (“undruggable” proteins).^285^ The same tethering concept is of course also useful to stabilize protein–protein interactions (PPIs).^286^

Nanopore sequencing technology is now an established method in the next-generation sequencing (NGS) toolbox.^287−289^ Recently, there has been even a nanopore reported that can discriminate all 20 cAAs including its PTMs.^290^ While nanopore sequencing is already useful, there is still room for improvement in various areas. For example, challenges such as low accuracy in certain fragment reads persist, leading to higher error rates compared to short-read NGS methods.^289^ The exploration of GCE to improve nanopore sequencing, by incorporating ncAAs into nanopores, represents a promising avenue for enhancing sensing properties and overcoming specific limitations in nanopore sequencing technologies. For example, proof-of-principle studies were carried out in which ncAAs were incorporated into nanopores and these nanopores showed unique sensing properties.^291,292^

While most GCE OTS are used on the protein translation level to incorporate one or multiple ncAAs, Kato showed that the PylRS can be used on the transcriptional level as well to create synthetic translational switches.^293^ The use of ncAA substrate **22** in this context suggests the potential for developing synthetic regulatory mechanisms that modulate translation based on transcriptional signals. This approach expands the toolkit of synthetic biology and provides additional means for designing intricate control systems within cellular processes.

## 7 Expanding the Scope of OTS by Reassigning Sense Codons and Designing Codon Sizes

While the primary focus of the Review centers on PylRS OTS, this section aims to briefly address two pivotal challenges in orthogonal translation that bear general significance for the field’s future trajectory. The first challenge is that an increase in the number of codons, not just stop codons, that are used for ncAA incorporation correlates with an overall reduction in the efficiency of orthogonal translation.^27,56^ In section 4, it was discussed how that could be improved in regard to increasing the catalytic efficiency.

The second challenge is the limited number of ncAAs that can be simultaneously incorporated. The field made significant progress in increasing the number of “empty” codons by creating a specific *E. coli* strain^115^ with three free ones, one amber termination codon and two Ser codons were freed up. While these improvements are great for all sorts of applications, the ultimate goal of encoding any number and kind of ncAA is still a long way away. Therefore, it is imperative for the field to advance toward systems enabling the permanent reassignment of more sense codons, facilitating the incorporation of virtually unlimited numbers and types of ncAAs for genetically encoded biopolymer syntheses.

Solving these intricate challenges is essential, as it promises not only to enhance the usability of GCE systems but also to significantly broaden the overall scope of OTS. All the ideas deliberated here hold crucial importance for the utilization of the PylRS system, currently deemed the most promising tool for GCE. However, we acknowledge that future identification and design of alternative systems may emerge to successfully overcome the discussed limitations.

### 7.1 Opportunities and Obstacles of Extending the Codon Size for GCE

Unfortunately, there are no natural free triplet codons, besides in a few engineered *E. coli* strains.^113−115^ Also a project is ongoing trying to free up the amber codon for yeast.^294^ So while there was some progress in the last years we will discuss this subject in a general manner. Within the triplet code, only three “blank” codons are available for genetic code expansion. However, for incorporating more than three kinds of ncAAs into a specific protein, the assignment of an extra codon is needed.

An interesting solution might involve the design of a platform that operates with quadruplet^295^ or even pentaplet codons. It is known for a long time now that quadruplet decoding happens in nature and might be even programmable.^296^ In earlier studies, Magliery, Anderson, and Schultz investigated codon and anticodon size constraints and found that tRNA serves as a “molecular ruler” that measures codon size during translation.^297^ Their results show that the translation apparatus enables the decoding of three-, four-, or five-based codons, with each codon type showing a preference for different sized tRNAs. They concluded that there are limits to both the size of codons, which is likely determined at the ribosome, and the size of the tRNA-anticodon loop, which corresponds to codon length. Unfortunately, quadruplet decoding is hampered by low efficiency because there is competition with triplet decoding.^295,298^ It is likely to persist in this state until comprehensive ribosomal engineering is undertaken to accommodate this type of decoding.

To date, up to four different ncAAs could be simultaneously incorporated into a protein using quadruplet codons.^298^ However, special 5′ UTR had to be engineered for that making this a specific application. The general use of quadruplets seems therefore likely to be constrained, also because cells with a new genetic code containing 256 quadruplets would pose challenges in terms of metabolic burden and code degeneracy, as discussed recently.^299^ OTS based on quadruplet codes or even the use of noncanonical base pairs could potentially be developed as an *in vitro* or *in vivo* platform which can operate as a subsystem (like a virtual machine in IT) for biopolymer biosynthesis.^300^ For *in vivo* platforms, it is conceivable that engineered eukaryotic host cells could enable that by harnessing their compartmentalization capabilities.^301^

### 7.2 Breaking the Degeneracy of the Triplet Code–Sense Codon Reassignment for OTS

The normal triplet code exhibits high degeneracy,^302^ and a promising approach to reprogramming the translation of ribosomal proteins in a broader context may involve breaking this degeneracy^303^ through the reassignment of sense codons to ncAAs. Early *in vitro* works by Hartman and colleagues in 2007 demonstrated that within the framework of the triplet code, there is significant potential for integrating new amino acids into the genetic code.^304^ Their work on *in vitro* ribosome-mediated translation showcased the insertion of 50 diverse noncanonical amino acids into peptide sequences through the native translation apparatus. Most of these ncAAs could later be incorporated *in vivo* by stop codon mediated incorporation, as shown in this Review. This strategy allows for the reassignment of approximately 70% of codons, leveraging the substrate tolerance of aaRS, a feature that highlights the adaptive promiscuity of these enzymes.^305^ Krishnakumar and co-workers have estimated that 30 to 40 sense codons are sufficient to encode the genetic information on an organism, leaving a considerable number of sense codons (>20) available for recoding with ncAAs.^306^

The experimental approach to addressing these challenges is nontrivial, given the difficulty in predicting which sense codons are most suitable for reassignment and determining the optimal orthogonal translation machinery for each codon.^307^ In 2014, our group attempted to exploit the degeneracy of the genetic code by freeing rare sense AUA codons from their original coding role and permanently reprogramming them to code for specific ncAA.^308^ Key to these efforts is that endogenous tRNA modifications can be used to achieve the expansion of the genetic code.^309^ Sakamoto and co-workers were the first to exploit the anticodon blindness of the tRNA^Pyl^ and showed that the rare arginine codon AGG could be assigned to l-homoarginine using PylRS OTS, which works with an orthogonal tRNA capable of decoding the rare codon.^31^ In a very recent study, Ding, Yu and Chen showed that sense codon reassignment can be done very efficiently in mammalian cells with the help of synthetic organelles. With this strategy, they encoded a total number of four different ncAAs into a target protein.^314^ We want to highlight that the PylRS system could play a vital role to further exend these approaches since the anticodon of tRNA^Pyl^ is freely programmable, in contrast to other OTS.

In an additional breakthrough, Hartman and colleagues conducted *in vitro* translation experiments, successfully assigning five different amino acids to the 6-fold degenerate leucine codon box and two different amino acids to the 4-fold degenerate valine codon box in a single experiment.^310^ These remarkable results demonstrated that the 10 leucine and valine codons could collectively encode for seven different amino acids, highlighting the extensive potential within the genetic code’s degeneracy. The next step is to adapt this protocol for orthogonal translation with ncAAs in living cells.

## 8 Conclusions and Outlook

The field of genetic code engineering has indeed made significant strides since its establishment nearly 30 years ago. Through reprogrammed protein translation, especially with the widespread use of *in vivo* methods based on stop-codon suppression, we have surpassed the natural repertoire of amino acids by at least 1 order of magnitude. The PylRS OTS alone has facilitated the incorporation of at least 342 ncAAs into proteins *in vivo*. This remarkable progress has opened new possibilities for expanding the chemical diversity of proteins and has broad implications for various applications, including biotechnology, medicine, and material science.

With such systems in hand, we have very sophisticated tools to rationally engineer bioactive peptides, protein scaffolds, complexes, and even whole proteomes by introducing various chemistries, well-known to organic syntheses, via ncAAs into life. These ncAAs bring many advantages over classical chemical or recombinant approaches as they enable: (i) the ability to genetically encode desired ncAAs and position them in the protein sequence allows precise control over the functionalization of biomolecules; (ii) *in vivo* production, where the ncAAs themselves can be produced intracellularly and subsequently inserted into target proteins or cell parts; (iii) the targeted functionalization, including site-directed bioconjugations, light-induced cross-linking, metal or cofactor binding, fluorophore or pharmacophore attachment, and adhesiveness, to name but a few.

Other crucial requirements to expand the scope of protein synthesis with ncAAs include cellular uptake, their intracellular metabolic stability, and translational activity (i.e., incorporation). Finally, it is necessary to reassign or expand coding triplets in the genetic code to insert ncAAs in target protein/peptide sequences. In this way, the integration of ncAAs into biological systems offers unprecedented opportunities for the design and manipulation of biomolecules with a degree of precision and versatility that cannot be achieved with classical chemical or recombinant approaches.

The fundamental methodological breakthrough in the field is that the problem of substrate specificity of orthogonal enzymes has finally been solved largely with the help of the PylRS system, as it is now possible to incorporate almost all types of amino acid substrates (natural and unnatural, long/heavy/bulky, aliphatic, aromatic, halogenated, bioorthogonal, etc.) into recombinant proteins. The yet untapped possibilities of ncAA incorporation will undoubtedly attract the interest of researchers from various fields such as machine learning, biophysics, biomedicine, and evolutionary biology.

Improving the robustness of such platforms can be achieved through genomic recordings and evolutionary methods such as adaptive laboratory evolution (ALE)^311^ or phage-assisted continuous evolution (PACE, discussed in section 4). Furthermore, the future direction of this field depends on the fusion of orthogonal translation with synthetic metabolism. This strategy aims to reduce the need for expensive external supplements of ncAAs by designing a synthetic metabolism that can work effectively with these various unnatural substrates (discussed in section 5).

PylRS-based OTSs on plasmid vectors pose a challenge in large-scale fermentations in all cell hosts. In such conditions, plasmid-free cells may become dominant during fermentation, presenting issues for biotechnological applications.^312^ Thus, maintaining a consistent gene dosage for the OTS component is essential to ensure effective protein synthesis and the sustained resilience of host cells across diverse environments. Therefore, we and others have recently demonstrated how genome editing tools are capable of simultaneously and safely inserting orthogonal pairs and other OTS-related genes as well as selected metabolic markers into the genome of a selected cell chassis.^141,142^ We believe that maintaining OTS systems extrachromosomally poses a substantial hurdle to transferring this technology to industrially relevant settings, given challenges such as vector instability, metabolic stress, and conflicts in replication control. Therefore, transitioning from extrachromosomal genes to robust genomically integrated OTS systems presents a promising avenue for designing suitable platforms for biotechnology built on expanded genetic codes.

The above elaborations indicate a clear trend in PylRS-based OTS research toward systems bioengineering. This requires a comprehensive understanding of how individual components such as the engineered aaRS-tRNA pairs function, both independently (e.g., *in vitro*) and integrated into the broader, highly systemic context of the cell (*in vivo*). For example, Fan, Söll, and co-workers demonstrated that certain mutated PylRS-tRNA^Pyl^ pairs exhibit no significant differences in kinetic parameters (*K*_M_, *k*_cat_). However, when these components are transferred into living cells, the performance of OTS improves significantly (up to 3-fold).^315^ This shows that looking only at the catalytic parameters in isolated *in vitro* reactions, which do not mimic the complicated intracellular environment, provides only limited insights. Instead, there is an urgent need to decipher how the properties of the individual components relate to the overall performance of the system in a cellular environment. Currently, our understanding in this area is largely based on empirical observations.

However, the increasing availability of vast biological databases containing extensive DNA and protein information offers promising opportunities for modeling materials, living cells, and even organisms. Utilizing these resources could facilitate the development of efficient OTS. Cells equipped with such OTSs and other orthogonal processes and components can be compared to microbial production units or chemical machines. They function similarly to Turing machines and von Neumann’s self-replicating automata and are able to control complex biochemical processes with precision and efficiency.^313^

Research teams worldwide are diligently developing technologies to create synthetic organisms capable of producing medically and industrially significant substances. This builds on humanity’s longstanding efforts to modify natural organisms, with direct genetic manipulation since the 1970s significantly boosting our capabilities. Consequently, the gap between modified and natural organisms is widening. The pursuit of orthogonalization of cellular processes suggests a future where genetically and metabolically distinct artificial life forms will emerge. These organisms would be genetically isolated production units that are safely confined to controlled laboratory conditions and have no chance of survival outside this environment.

## Supporting Information Available

All ncAAs with corresponding numbers, chemical structures as graphic and SMILES, PylRS constructs that can incorporate these ncAAs, including derived organism and mutations, references, and comments (XLSX)

## Abbreviations

5′ UTR: five prime untranslated region
aaRS: aminoacyl tRNA synthetase
ADC: antibody–drug conjugate
AMP: antimicrobial peptide
ALE: adaptive laboratory evolution
ASR: ancestral sequence reconstruction
BocK: *N*^*ε*^-*tert*-butoxycarbonyl-l-lysine
cAA: canonical amino acid
chPylRS: chimeric PylRS
CuAAC: copper(I)-catalyzed alkyne–azide cycloaddition
CTD: C-terminal domain
DBCO: dibenzocyclooctyne
DMAP: 4-(dimethylamino)pyridine
DSF: differential scanning fluorimetry
EPR: electron paramagnetic resonance
FRET: Förster resonance energy transfer
GCE: genetic code expansion
GPCR: G protein-coupled receptor
HER2: human epidermal growth factor receptor 2
HGT: horizontal gene transfer
HisRS: histidyl-tRNA synthetase
IEDDA: inverse electron demand Diels–Alder
IL-2: interleukin 2
IleRS: isoleucyl-tRNA synthetase
IR: infrared spectroscopy
LeuRS: leucyl-tRNA synthetase
LPMO: lytic polysaccharide monooxygenases
LUCA: last universal common ancestor
MetRS: methionyl-tRNA synthetase
NES: nuclear export signal
NGS: next-generation sequencing
NLS: nuclear localization signal
NP: natural product
NPC: nascent polypeptide chain
ncAA: noncanonical amino acid
NMR: nuclear magnetic resonance
NTD: N-terminal domain
OGT: optimal growth temperature
OTS: orthogonal translation system
PACE: phage-assisted continuous evolution
PANCE: phage-assisted noncontinuous evolution
PD-1: programmed cell death protein-1
PD-L1: programmed cell death-ligand 1
PERx: proximity-enabled reactive therapeutics
PPi: pyrophosphate
PPI: protein–protein interaction
PTC: premature termination codon
PTM: posttranslational modifications
Pyl: pyrrolysine
PylRS: pyrrolysyl-tRNA synthetase
RF: release factor
RF 1: release factor 1
scFv: single-chain variable fragment
SCS: stop codon suppression
SmbP: small metal binding protein
SPAAC: strain-promoted alkyne–azide cycloaddition
TyrRS: tyrosyl-tRNA synthetase
